# Supplementary material for: Structure and Characterization of Phosphoglucomutase 5 from Atlantic and Baltic Herring—An Inactive Enzyme with Intact Substrate Binding
Source: Biomolecules. 2020 Dec 3;10(12):1631. doi: 10.3390/biom10121631 (PMC7761743; doi:10.3390/biom10121631)
Supplement: Supplementary file 1 [file biomolecules-10-01631-s001.pdf]

Supplementary information for:

Structure and characterization of phosphoglucomutase 5 from herring - an inactive enzyme with intact substrate binding

Robert Gustafsson [1,†], Ulrich Eckhard [1, †,‡], Weihua Ye [2], Erik D. Enbody [2], Mats Pettersson [2], Per Jemth [2], Leif Andersson [2,3,4] and Maria Selmer [1,*]

[1] Department of Cell and Molecular Biology, Uppsala University, BMC, Box 596, SE-751 24 Uppsala, Sweden; robert.gustafsson@icm.uu.se (R.G.); ueccri@ibmb.csic.es (U.E.)

[2] Department of Medical Biochemistry and Microbiology, Uppsala University, BMC, Box 582, SE-751 23 Uppsala, Sweden; weihua.ye@imbim.uu.se (W.Y.); erik.enbody@imbim.uu.se (E.D.E.); mats.pettersson@imbim.uu.se (M.P.); per.jemth@imbim.uu.se (P.J.); Leif.Andersson@imbim.uu.se (L.A.)

[3] Department of Veterinary Integrative Biosciences, Texas A&M University, College Station, TX 77843, USA

[4] Department of Animal Breeding and Genetics, Swedish University of Agricultural Sciences,
SE-75007 Uppsala, Sweden

* Correspondence: maria.selmer@icm.uu.se

† These authors contributed equally.

‡ Current address: Proteolysis Lab, Department of Structural Biology, Molecular Biology Institute of Barcelona, CSIC, Barcelona Science Park, Baldiri Reixac, 15-21, 08028 Barcelona, Catalonia, Spain

Content:

Figure S1-S8

Table S1-S5

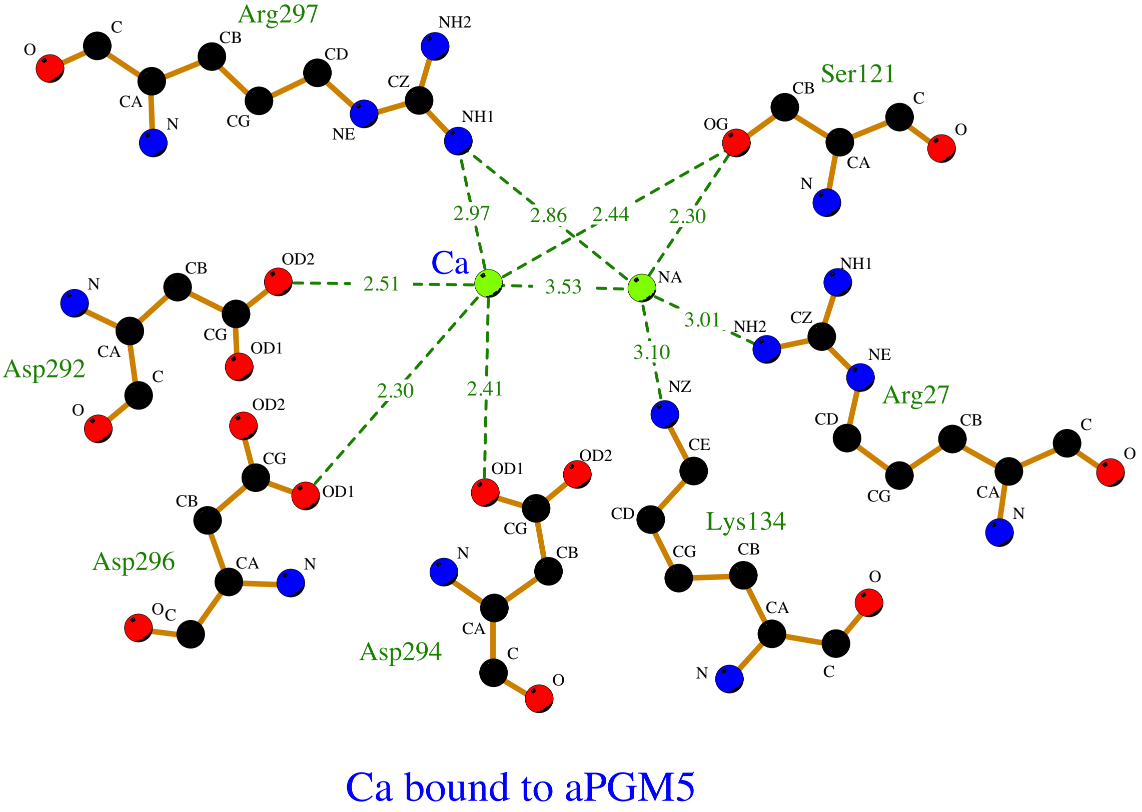

Figure S1: Coordination of Ca^{2+} in apo aPGM5. Ca^{2+} and aPGM5 residues are labelled blue and green, respectively. Green dashed lines indicate hydrogen bonds or electrostatic interactions, with distances indicated. Ca^{2+} and sodium ion are shown as green spheres. Figure prepared using LigPlot+ [1].

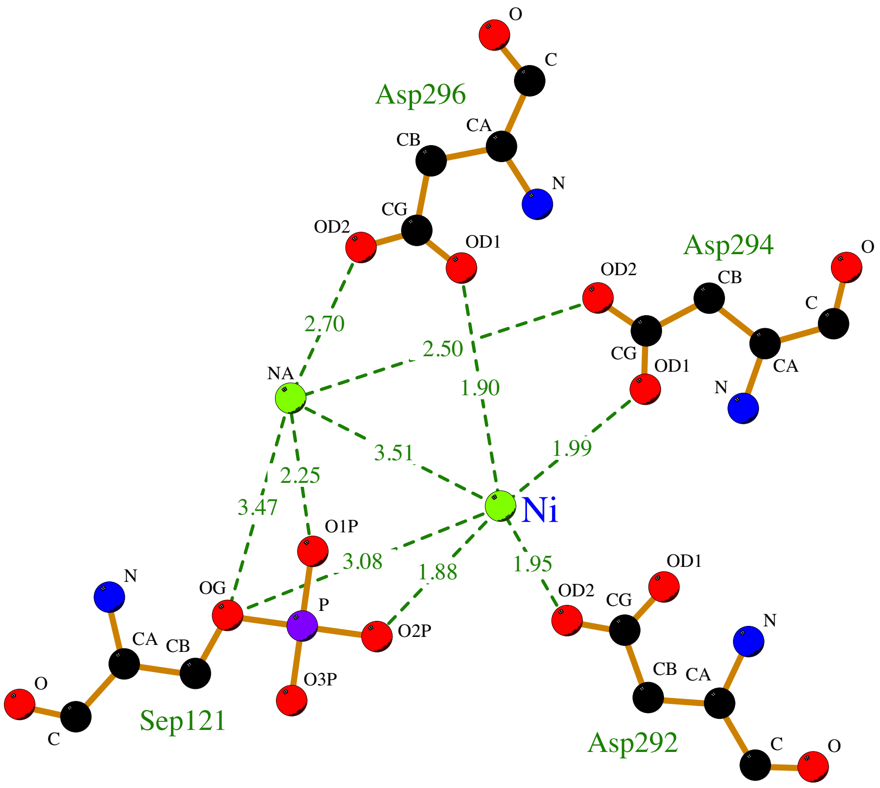

Ni bound to bPGM5 apo

Figure S2: Coordination of Ni^{2+} in apo bPGM5. Ni^{2+} and bPGM5 residues are labelled blue and green, respectively. Green dashed lines indicate hydrogen bonds or electrostatic interactions, with distances indicated. Ni^{2+} and sodium ion are shown as green spheres. Figure prepared using LigPlot+ [1].

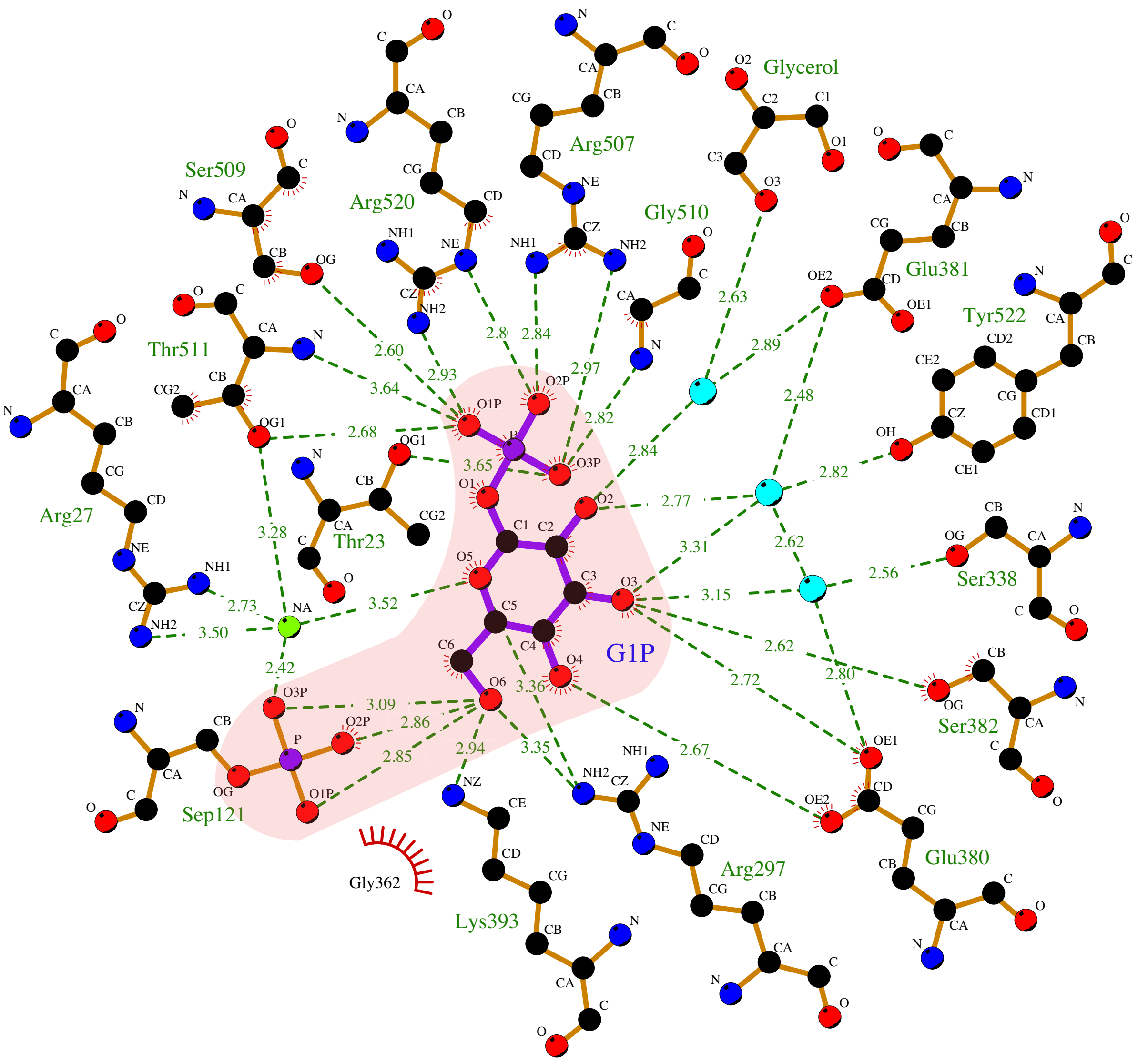

G1P bound to bPGM5

Figure S3: Coordination of G1P in bPGM5. G1P and bPGM5 residues are labelled blue and green, respectively. Green dashed lines indicate hydrogen bonds or electrostatic interactions, with distances indicated. Residues or atoms involved in hydrophobic interactions are represented by an arc with spokes radiating towards the binding partners they contact. Bridging waters are shown as blue spheres, and sodium ion shown as green sphere. Figure prepared using LigPlot+ [1].

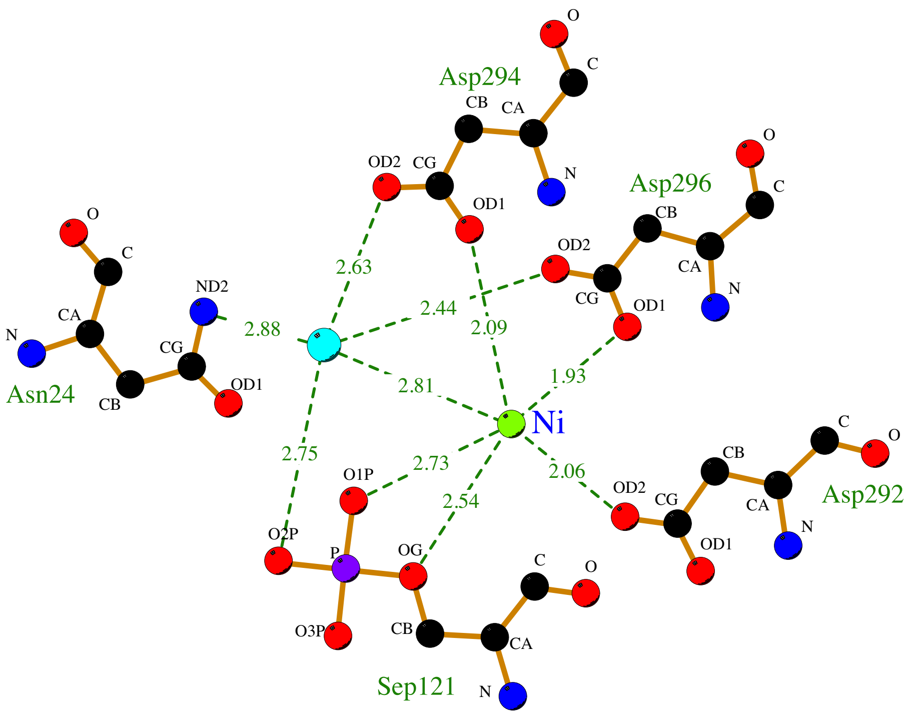

Ni bound to bPGM5

Figure S4: Coordination of Ni^{2+} in the bPGM5 G1P complex. Ni^{2+} and bPGM5 residues are labelled blue and green, respectively. Green dashed lines indicate hydrogen bonds or electrostatic interactions, with distances indicated. Bridging water is shown as blue sphere. Figure prepared using LigPlot+ [1].

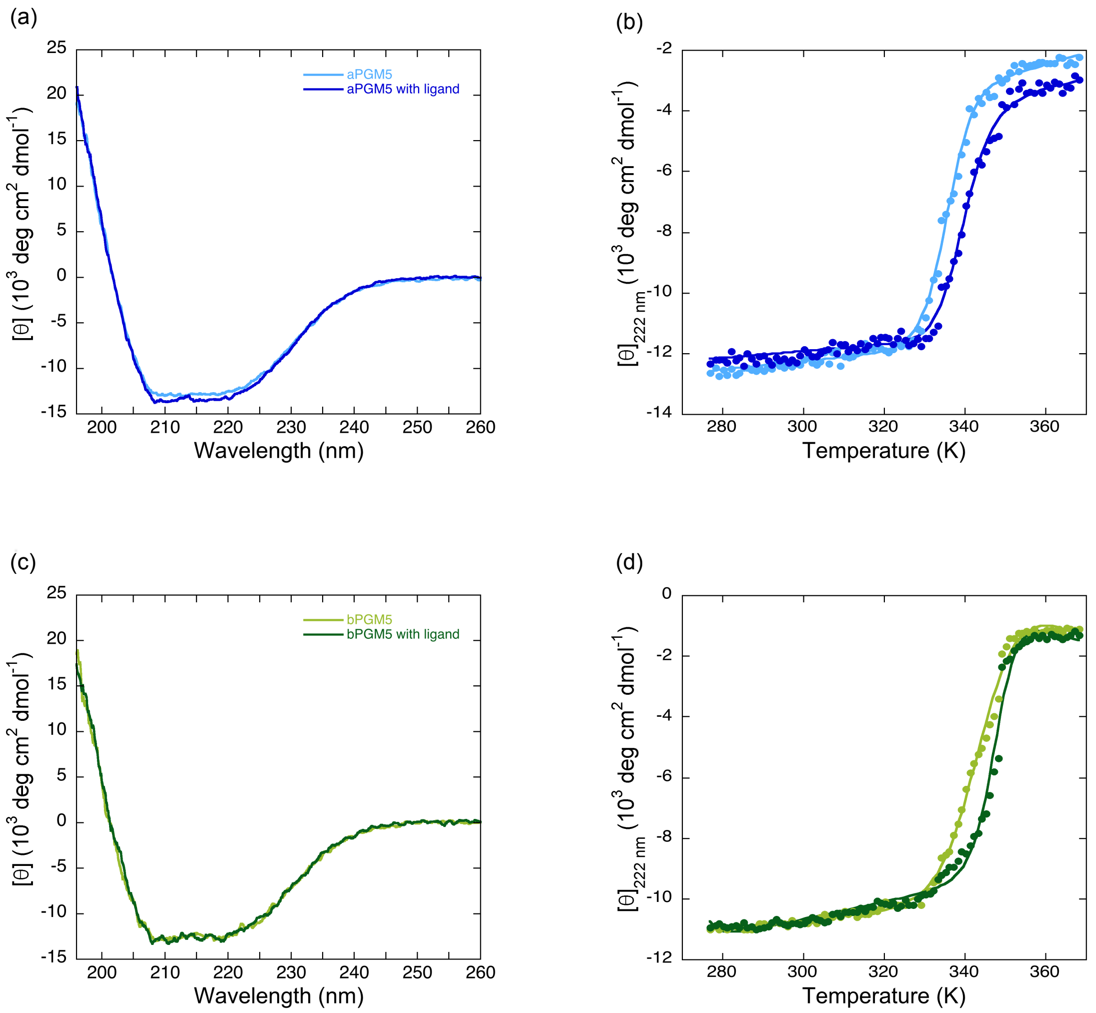

Figure S5: **CD spectroscopy measurements.** CD spectroscopy measurements for aPGM5 (**(a)** and **(b)**, blue) and bPGM5 (**(c)** and **(d)**, green) in the absence of ligand (light color) and in the presence of ligand, 0.5 mM G16P (dark color). (**(a)** and **(c)**) Representative CD spectra for aPGM5 **(a)** and bPGM5 **(c)** at 25 °C. (**(b)** and **(d)**) Representative thermal denaturation curves for aPGM5 **(b)** and bPGM5 **(d)** monitored at 222 nm and the T_m values are 336 K (aPGM5 apo), 340 K (aPGM5 with ligand), 342 K (bPGM5 apo) and 346 K (bPGM5 with ligand), respectively.

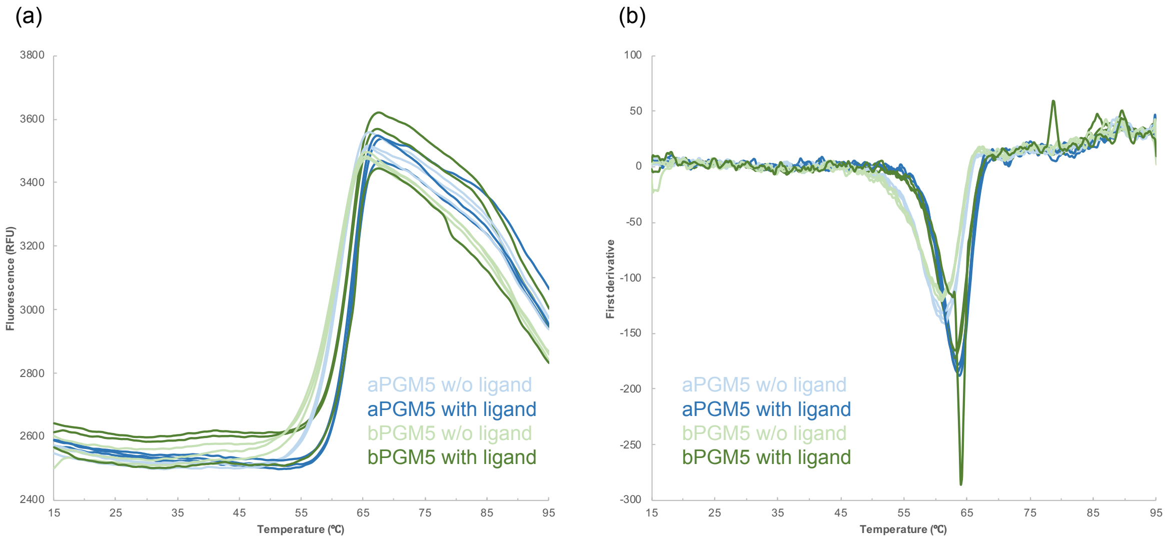

Figure S6: DSF data. DSF melting curves **(a)** and first derivative **(b)** for aPGM5 (blue) and bPGM5 (green) without His6-tag, measured without (light color) and in presence of (dark color) 500 µM reaction intermediate G16P.

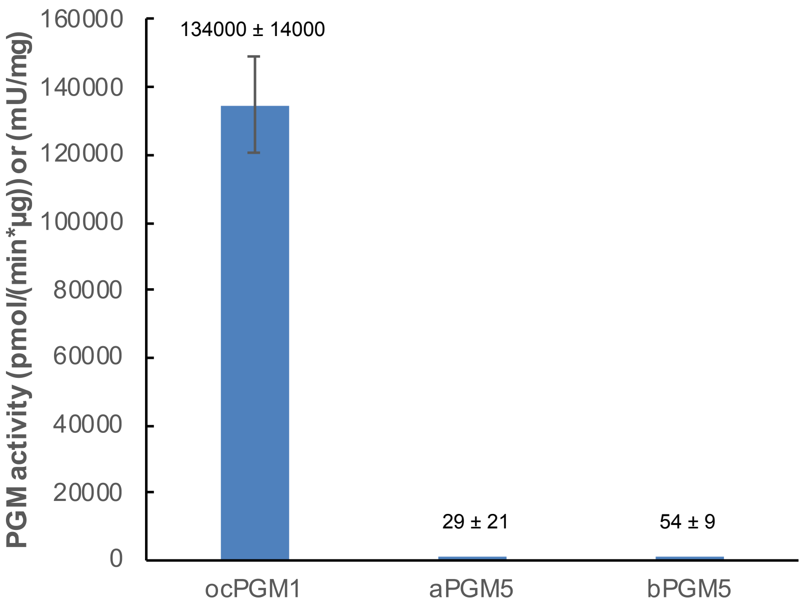

Figure S7: **Enzymatic activity data.** Phosphoglucomutase activity assay of positive control rabbit muscle PGM1 (ocPGM1), aPGM5 and bPGM5 in presence of $MgCl_2$ and G16P. Results are shown as average with error bars of +/- one standard deviation from triplicate experiments after subtraction of background activity. Background activity was measured without addition of substrate.

Figure S8: Multiple sequence alignment of PGM5 and PGM1 from 10 fish species and 10 mammals. The alignment was done using Clustal Omega and visualized using ESPript [2].

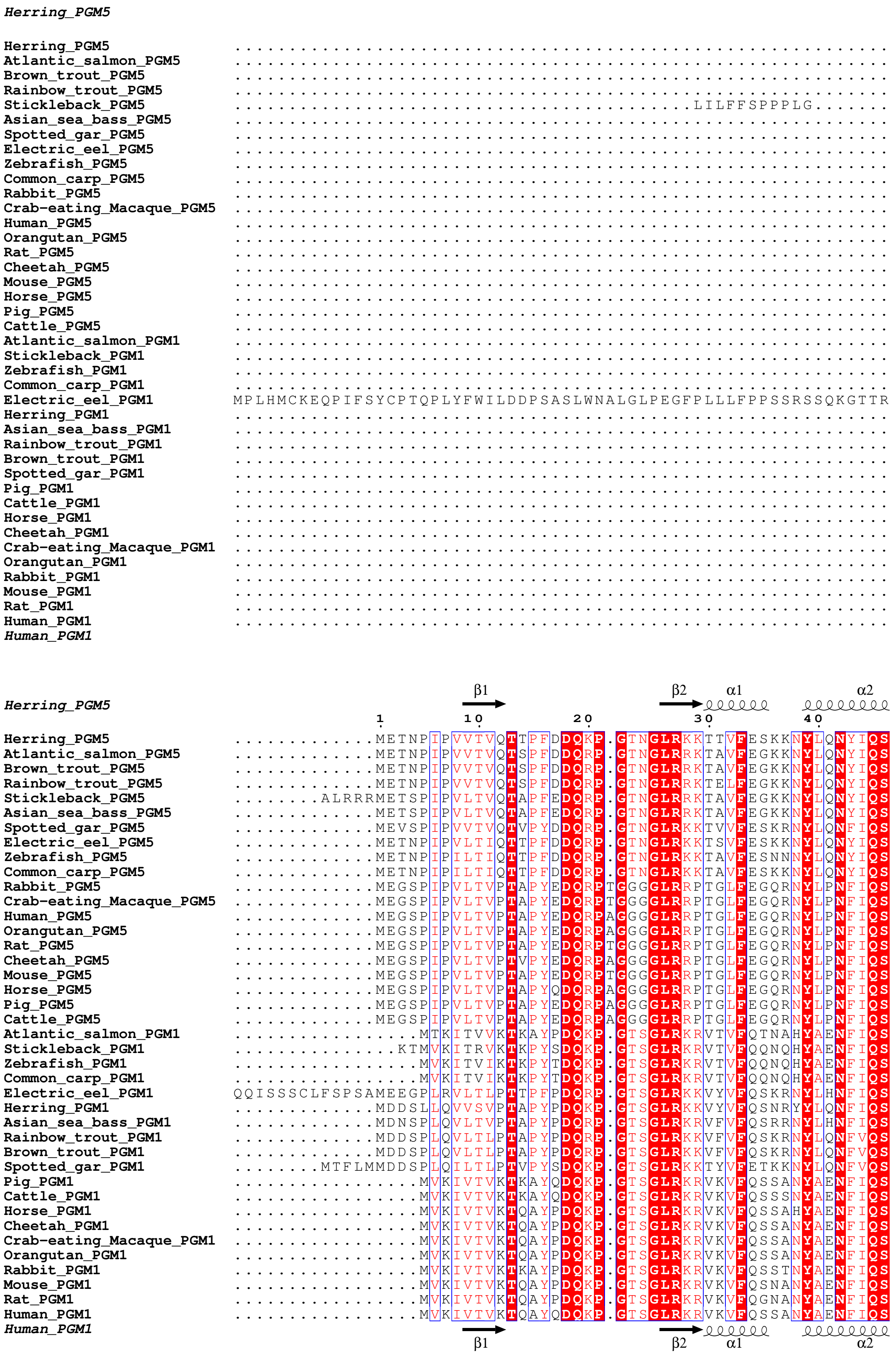
Herring_PGM5
Herring_PGM5
Atlantic_salmon_PGM5
Brown_trout_PGM5
Rainbow_trout_PGM5
Stickleback_PGM5 LILFFSPPPLG
Asian_sea_bass_PGM5
Spotted_gar_PGM5
Electric_eel_PGM5
Zebrafish_PGM5
Common_carp_PGM5
Rabbit_PGM5
Crab-eating_Macaque_PGM5
Human_PGM5
Orangutan_PGM5
Rat_PGM5
Cheetah_PGM5
Mouse_PGM5
Horse_PGM5
Pig_PGM5
Cattle_PGM5
Atlantic_salmon_PGM1
Stickleback_PGM1
Zebrafish_PGM1
Common_carp_PGM1
Electric_eel_PGM1 MPLHMCKEQPIFSYCPTQPLYFWILDDPSASLWNALGLPEGFPLLLFPPSSRSSQKGTTR
Herring_PGM1
Asian_sea_bass_PGM1
Rainbow_trout_PGM1
Brown_trout_PGM1
Spotted_gar_PGM1
Pig_PGM1
Cattle_PGM1
Horse_PGM1
Cheetah_PGM1
Crab-eating_Macaque_PGM1
Orangutan_PGM1
Rabbit_PGM1
Mouse_PGM1
Rat_PGM1
Human_PGM1
Human_PGM1
β1 β2 α1 α2
Herring_PGM5
1 10 20 30 40
Herring_PGM5 METNPIPVVTVQTTPFDDQKP.GTNGLRKKTTVFESKKNYLQNYIQS
Atlantic_salmon_PGM5 METNPIPVVTVQTSPFDDQRP.GTNGLRKKTAVFEGKKNYLQNYIQS
Brown_trout_PGM5 METNPIPVVTVQTSPFDDQRP.GTNGLRKKTAVFEGKKNYLQNYIQS
Rainbow_trout_PGM5 METNPIPVVTVQTSPFDDQRP.GTNGLRKKTELFEGKKNYLQNYIQS
Stickleback_PGM5 ALRRRMETSPIPVLTVQTAPFEDQRP.GTNGLRKKTAVFEGKKNYLQNYIQS
Asian_sea_bass_PGM5 METSPIPVLTVQTAPFEDQRP.GTNGLRKKTAVFEGKKNYLQNYIQS
Spotted_gar_PGM5 MEVSPIPVVTVQTVPYDDQRP.GTNGLRKKTVVFESKRNYLQNFIQS
Electric_eel_PGM5 METNPIPVLTIQTTPFDDQRP.GTNGLRKKTSVFESKKNYLQNYIQS
Zebrafish_PGM5 METNPIPILTIQTTPFDDQRP.GTNGLRKKTAVFESNNNYLQNYIQS
Common_carp_PGM5 METNPIPILTIQTTPFDDQRP.GTNGLRKKTAVFESKKNYLQNYIQS
Rabbit_PGM5 MEGSPIPVLTVPTAPYEDQRPTGGGGLRRPTGLFEGQRNYLPNFIQS
Crab-eating_Macaque_PGM5 MEGSPIPVLTVPTAPYEDQRPTGGGGLRRPTGLFEGQRNYLPNFIQS
Human_PGM5 MEGSPIPVLTVPTAPYEDQRPAGGGGLRRPTGLFEGQRNYLPNFIQS
Orangutan_PGM5 MEGSPIPVLTVPTAPYEDQRPAGGGGLRRPTGLFEGQRNYLPNFIQS
Rat_PGM5 MEGSPIPVLTVPTAPYEDQRPTGGGGLRRPTGLFEGQRNYLPNFIQS
Cheetah_PGM5 MEGSPIPVLTVPTVPYEDQRPAGGGGLRRPTGLFEGQRNYLPNFIQS
Mouse_PGM5 MEGSPIPVLTVPTAPYEDQRPTGGGGLRRPTGLFEGQRNYLPNFIQS
Horse_PGM5 MEGSPIPVLTVPTAPYQDQRPAGGGGLRRPTGLFEGQRNYLPNFIQS
Pig_PGM5 MEGSPIPVLTVPTAPYEDQRPAGGGGLRRPTGLFEGQRNYLPNFIQS
Cattle_PGM5 MEGSPIPVLTVPTAPYEDQRPAGGGGLRRPTGLFEGQRNYLPNFIQS
Atlantic_salmon_PGM1 MTKITVVKTKAYPDQKP.GTSGLRKRVTVFQTNAHYAENFIQS
Stickleback_PGM1 KTMVKITRVKTKPYSDQKP.GTSGLRKRVTVFQQNQHYAENFIQS
Zebrafish_PGM1 MVKITVIKTKPYTDQKP.GTSGLRKRVTVFQQNQHYAENFIQS
Common_carp_PGM1 MVKITVIKTKPYTDQKP.GTSGLRKRVTVFQQNQHYAENFIQS
Electric_eel_PGM1 QQISSSCLFSPSAMEEGPLRVLTLPTTPFPDQRP.GTSGLRKKVYVFQSKRNYLHNFIQS
Herring_PGM1 MDDSLLQVVSVPTAPYPDQKP.GTSGLRKKVYVFQSNRYYLQNFIQS
Asian_sea_bass_PGM1 MDNSPLQVLTVPTAPYPDQRP.GTSGLRKRVFVFQSRRNYLHNFIQS
Rainbow_trout_PGM1 MDDSPLQVLTLPTAPYPDQRP.GTSGLRKKVFVFQSKRNYLQNFVQS
Brown_trout_PGM1 MDDSPLQVLTLPTAPYPDQRP.GTSGLRKKVFVFQSKRNYLQNFVQS
Spotted_gar_PGM1 MTFLMMDDSPLQILTLPTVPYSDQKP.GTSGLRKKTYVFETKKNYLQNFVQS
Pig_PGM1 MVKIVTVKTKAYQDQKP.GTSGLRKRVKVFQSSANYAENFIQS
Cattle_PGM1 MVKIVTVKTKAYQDQKP.GTSGLRKRVKVFQSSSNYAENFIQS
Horse_PGM1 MVKIVTVKTQAYPDQKP.GTSGLRKRVKVFQSSAHYAENFIQS
Cheetah_PGM1 MVKIVTVKTQAYPDQKP.GTSGLRKRVKVFQSSANYAENFIQS
Crab-eating_Macaque_PGM1 MVKIVTVKTQAYPDQKP.GTSGLRKRVKVFQSSANYAENFIQS
Orangutan_PGM1 MVKIVTVKTQAYPDQKP.GTSGLRKRVKVFQSSANYAENFIQS
Rabbit_PGM1 MVKIVTVKTKAYPDQKP.GTSGLRKRVKVFQSSTNYAENFIQS
Mouse_PGM1 MVKIVTVKTQAYPDQKP.GTSGLRKRVKVFQSNANYAENFIQS
Rat_PGM1 MVKIVTVKTQAYPDQKP.GTSGLRKRVKVFQGNANYAENFIQS
Human_PGM1 MVKIVTVKTQAYQDQKP.GTSGLRKRVKVFQSSANYAENFIQS
Human_PGM1
β1 β2 α1 α2

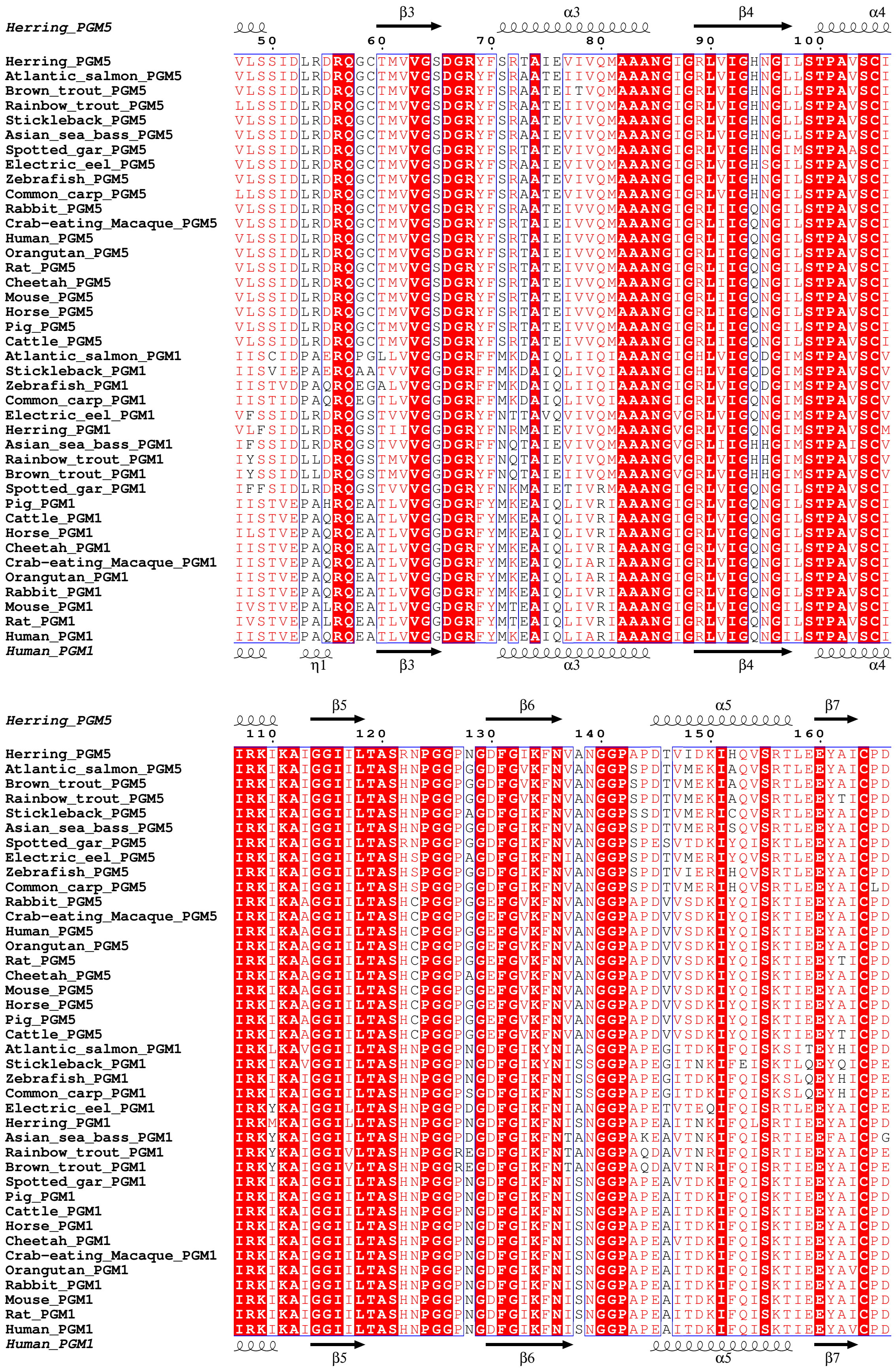
Herring_PGM5
β3
α3
β4
α4
50
60
70
80
90
100
Herring_PGM5
Atlantic_salmon_PGM5
Brown_trout_PGM5
Rainbow_trout_PGM5
Stickleback_PGM5
Asian_sea_bass_PGM5
Spotted_gar_PGM5
Electric_eel_PGM5
Zebrafish_PGM5
Common_carp_PGM5
Rabbit_PGM5
Crab-eating_Macaque_PGM5
Human_PGM5
Orangutan_PGM5
Rat_PGM5
Cheetah_PGM5
Mouse_PGM5
Horse_PGM5
Pig_PGM5
Cattle_PGM5
Atlantic_salmon_PGM1
Stickleback_PGM1
Zebrafish_PGM1
Common_carp_PGM1
Electric_eel_PGM1
Herring_PGM1
Asian_sea_bass_PGM1
Rainbow_trout_PGM1
Brown_trout_PGM1
Spotted_gar_PGM1
Pig_PGM1
Cattle_PGM1
Horse_PGM1
Cheetah_PGM1
Crab-eating_Macaque_PGM1
Orangutan_PGM1
Rabbit_PGM1
Mouse_PGM1
Rat_PGM1
Human_PGM1
Human_PGM1
η1
β3
α3
β4
α4
Herring_PGM5
β5
β6
α5
β7
110
120
130
140
150
160
Human_PGM1
β5
β6
α5
β7

```
                                    β8          β9          α6          α7          β10
Herring_PGM5
                               170       180       190       200       210       220
Herring_PGM5                 MRIDLSRLGRQDFDLENKFKPFRVEIVDSVEVYLNLLRGIFDFNAIKGLLTGPDQLKMRV
Atlantic_salmon_PGM5         LRIDLARLGRHDFDLENKFKPFRVEIVDSVELYLNLLRTIFDFNAIKSLLTGPEQLKIRI
Brown_trout_PGM5             LRIDLARLGRHDFDLENKFKPFRVEIVDSVELYLNLLRTIFDFNAIKSLLTGPEQLKIRI
Rainbow_trout_PGM5           LHIDLARLGRHDFDLENKFKPFRVEIVDSVEVYLNLLRTIFDFSAIKTLLTGPEQLKIRV
Stickleback_PGM5             LRIDLSRLGRQEFDLENKFKPFRVEIVDSVELYLQLLRNIFDFNAIKSLFSGPDQLKIHI
Asian_sea_bass_PGM5          LRIDLSRLGRQEFDLENKFKPFRVEIVDSVEVYLQLLRNIFDFNAIKSLLTGSNQLKIHI
Spotted_gar_PGM5             LRIDLARLGRQDFDLENKFKPFRVEIVDSVEIYLNMLRSIFDFSAIKGLLTGPDQLKIRI
Electric_eel_PGM5            LHIDLSRLGRQDFDLENKFKPFRVEIVDSVEIYLNMLRGIFDFGAIKSLLTGPEQLKIRI
Zebrafish_PGM5               LHIDLSRLGRQDFDLENKFKPFRVEIVDSVEIYFNLLRGIFDFSGIKSLLTGPNQLKIRI
Common_carp_PGM5             LHIDLSRLGRHDFDLENKFKPFRVEIVDSVEIYLNMLRGIFDFGAIKSLLTGPKQLKIRI
Rabbit_PGM5                  LRIDLSRLGRQEFDLENKFKPFRVEIVDPVDIYLNLLRTIFDFNAIKSLLTGPSQLKIRV
Crab-eating_Macaque_PGM5     LRIDLSRLGRQEFDLENKFKPFRVEIVDPVDIYLNLLRTIFDFHAIKSLLTGPSQLKIRV
Human_PGM5                   LRIDLSRLGRQEFDLENKFKPFRVEIVDPVDIYLNLLRTIFDFHAIKGLLTGPSQLKIRI
Orangutan_PGM5               LRIDLSRLGRQEFDLENKFKPFRVEIVDPVDIYLNLLRTIFDFHAIKSLLTGPSQLKIRI
Rat_PGM5                     LRIDLSRLGRQEFDLENKFKPFRVEIVDPVDIYLNLLRTIFDFNAIKSLLTGPSQLKIRV
Cheetah_PGM5                 LRIDLSRLGRQEFDLENKFKPFRVEIVDPVDIYLNLLRTIFDFNAIKSLLTGPGQLKIRV
Mouse_PGM5                   LRIDLSRLGRQEFDLENKFKPFRVEIVDPVDIYLNLLRNIFDFNAIKSLLTGPSQLKIRV
Horse_PGM5                   LRIDLSRLGRQEFDLENKFKPFRVEIVDPVDIYLNLLRTIFDFNAIKSLLTGPGQLKIRV
Pig_PGM5                     LRIDLSRLGRQEFDLENKFKPFRVEIVDPVDIYLNLLRTIFDFNAIKSLLTGPGQLKIRV
Cattle_PGM5                  LRIDLSRLGRQEFDLENKFKPFRVEIVDPVDIYLNLLRTIFDFNAIKSLLTGPGQLKIRV
Atlantic_salmon_PGM1         LKVDISKIGKQTFNVD.TFKPMTVEIVDSVEAYAEMLRGIFDFHALKGLVSGSNHIKVRL
Stickleback_PGM1             LKIDLSKIGKQTFEVD.TFKPFTVEIVDSVEAYAEMLRSIFNFAELKELLSGANHINVRL
Zebrafish_PGM1               LKVDLSTIGKQTFEVD.TFKPFTVEIVDSVESYAEMLRDIFDFAALKELLSGPNHINVRL
Common_carp_PGM1             LKVDLSATGKQIFEVD.TFKPFTVEIVDSVESYAEMLRDIFDFAALKELLSGPNHINVRL
Electric_eel_PGM1            LTVDLVTLGKQTFDLENKFKPFTVEIVDSVESYANMLRNIFDFAALKELLSGENHIRIRL
Herring_PGM1                 LRVDLATLGKQTFDLENKFKPFTVEIVDPVEAYANMLRNIFDFAALKEMLSGHNYIKIRL
Asian_sea_bass_PGM1          LRVDLLTLGKQTFDLENKFKPFTVEIVDSVESYANMLRNIFDFAALKELLSGENHIKIRI
Rainbow_trout_PGM1           IRVDLATLSKQTFDLENKFKPFTVEIVDSVESYANMLRNIFDFAALKELLSGENHIKIQL
Brown_trout_PGM1             LRVDLATLSKQKFDLENKFKPFTVEIVDSVESYANMLRNIFDFAALKELLSGENHIKIQL
Spotted_gar_PGM1             LKVDLTTIGKQQFDLENKFKPFTVEIIDSVESYANMLRNIFDFAALKELLSGQNQLKIRI
Pig_PGM1                     LKVDLGVLGKQQFDLENKFKPFTVEIVDSVEAYATMLRNIFDFNALKELLSGPNRLKIRI
Cattle_PGM1                  LHVDLGVLGKQQFDLENKFKPFTVEIVDSVEAYATMLRNIFDFNALKELLSGPNRLKIRI
Horse_PGM1                   LKVDLGVLGKQQFDLENKFKPFTVEIVDSVEAYATMLRNIFDFNALKELLSGPNRLKIRI
Cheetah_PGM1                 LKIDLGVLGKQQFDLENKFKPFTVEIVDSVEAYASMLRNIFDFNALKELLSGPNRLKIRI
Crab-eating_Macaque_PGM1     LKVDLGVLGKQQFDLENKFEPFTVEIVDSVEAYATMLRNIFDFSALKELLSGPNRLKIRI
Orangutan_PGM1               LKVDLGVLGKQQFDLENKFKPFTVEIVDSVEAYATMLRSIFDFSALKELLSGPNRLKIRI
Rabbit_PGM1                  LKVDLGVLGKQQFDLENKFKPFTVEIVDSVEAYATMLRNIFDFNALKELLSGPNRLKIRI
Mouse_PGM1                   LKVDLGVLGKQQFDLENKFKPFTVEIVDSVEAYATMLRNIFDFNALKELLSGPNRLKIRI
Rat_PGM1                     LKVDLGVLGKQQFDLENKFKPFTVEIVDSVEAYATMLRNIFDFNALKELLSGPNRLKIRI
Human_PGM1                   LKVDLGVLGKQQFDLENKFKPFTVEIVDSVEAYATMLRSIFDFSALKELLSGPNRLKIRI
Human_PGM1
                                    β8          β9          α6          α7          β10

                                    α8          η1 β11      η2                α9
Herring_PGM5
                               230       240       250       260       270       280
Herring_PGM5                 DAMSGVMGPYVRRILCDELGAPANSAVNCVPLEDFGGHYPDPNLTYATGLVDAMKGGEFG
Atlantic_salmon_PGM5         DAMNGVMGLYVRRIMCDELGAPANAAVNCVPMEDFGGRPPEPNLTYAAPLVDAMKGGDIG
Brown_trout_PGM5             DAMNGVMGLYVRRIMCDELGAPANAAVNCVPMEDFGGRPPEPNLTYAAPLVDAMKGGDIG
Rainbow_trout_PGM5           DAMNGVMGPYVRRILCDELGAPANAAVNCVPLEDFGGRPPEPSLTYAAPLVDAMKGGDFG
Stickleback_PGM5             DAMNGVMGPYVRRILCDELGAPANSAVNCVPLEDFGGRPPEPNLTYATSLVDGMKGGDFG
Asian_sea_bass_PGM5          DAMNGVMGPYVRRILCDELGAPANSAVNCVPLEDFGGRPPEPNLTYATSLVDAMKGGDFG
Spotted_gar_PGM5             DAMNGVMGPYVRRILCDELGAPANSAVSCVPLEDFGGQYPDPSLPYAASLVEAMKGGEFG
Electric_eel_PGM5            DAMNGVMGPYVRRILCDELGAPANSAVNCVPLEDFGGQHPNPNPTFAVSLVESMKGGEFG
Zebrafish_PGM5               DAMNGVMGPYVRRILCDELGAPANSAVNCVPLEDFGGQHPNPNPAFAGPLVDSMKGGEFG
Common_carp_PGM5             DAMNGVMGPYVRRILCDELGAPANSAVNCVPLEDFGGQHPNPNPAFAAALVDSMKXGEFG
Rabbit_PGM5                  DAMHGVMGPYVRKVLCDELGAPANSAINCVPLEDFGGQHPDPNLTYATTLLEAMKGGEYG
Crab-eating_Macaque_PGM5     DAMHGVMGPYVRKVLCDELGAPANSAINCVPLEDFGGQHPDPNLTYATTLLEAMKGGEYG
Human_PGM5                   DAMHGVMGPYVRKVLCDELGAPANSAINCVPLEDFGGQHPDPNLTYATTLLEAMKGGEYG
Orangutan_PGM5               DAMHGVMGPYVRKVLCDELGAPANSAINCVPLEDFGGQHPDPNLTYATTLLEAMKGGEYG
Rat_PGM5                     DAMHGVMGPYVRKVLCDELGAPANSAINCVPLEDFGGQHPDPNLTYATTLLEAMKGGEYG
Cheetah_PGM5                 DAMHGVMGPYVRKVLCDELGAPANSAINCVPLEDFGGQHPDPNLTYATTLLEAMKGGEYG
Mouse_PGM5                   DAMHGVMGPYVRKVLCDELGAPANSAINCVPLEDFGGQHPDPNLTYATTLLEAMKGGEYG
Horse_PGM5                   DAMHGVMGPYVRKVLCDELGAPANSAINCVPLEDFGGQHPDPNLTYATTLLEAMKGGEYG
Pig_PGM5                     DAMHGVMGPYVRKVLCDELGAPANSAINCVPLEDFGGQHPDPNLTYATTLLEAMKGGEYG
Cattle_PGM5                  DAMHGVMGPYVRKVLCDELGAPANSAINCVPLEDFGGQHPDPNLTYATTLLEAMKGGEYG
Atlantic_salmon_PGM1         DAMHGVVGPYVKKIVCEELGAAADGAFNCVPKEDFGGHHPDPNLTYASDLVNAMKGGEYD
Stickleback_PGM1             DAMHGVVGPYVRKIVCEELGSPAHSAVNCVPKEDFGGHHPDPNLTYAADLVNTMKGGEYD
Zebrafish_PGM1               DAMHGVVGPYVKKIVCEELGSPANSAVNCVPSEDFGGHHPDPNLTYAADLVNTMKGGEYD
Common_carp_PGM1             DAMHGVMGPYVRKIVCEELGSPANSAVNCVPSEDFGGHHPDPNLTYAADLVSAMKGGEYD
Electric_eel_PGM1            DAMHGVVGPYVKKILCEELGSPANSAINCIPLEDFGGQHPDPNLTHAADLVETMKSGQYD
Herring_PGM1                 DAMHGVMGPYLKRIMCEELGCPVNSVINCVPLEDFGGKHPDPNLTYAAELVEAMRSGQFD
Asian_sea_bass_PGM1          DAMHGAVGPYVKRILCEELGCPANSAINCVPVEDFGGQHPDPNLTYAADLVDSMKDGQYD
Rainbow_trout_PGM1           DAMHGVMGPYVKRILCEELGSPANSAINCVPLQDFGGQHPDPNLTYAADLVETMRGGQYD
Brown_trout_PGM1             DAMHGVMGPYVKRILCEELGSPANSAINCVPLQDFGGQHPDPNLTYAADLVETMRGGQYD
Spotted_gar_PGM1             DAMHGVVGPYVKKILCEELGSPANSAVNCVPLEDFGGHHPDPNLTYAADLVETMKGGEYD
Pig_PGM1                     DAMHGVVGPYVKKILCEELGAPANSAVNCVPLEDFGGHHPDPNLTYAADLVETMKTGEHD
Cattle_PGM1                  DAMHGVVGPYVKKILCEELGAPANSAVNCVPLEDFGGHHPDPNLTYAADLVETMKTGEHD
Horse_PGM1                   DAMHGVVGPYVKKILCEELGAPANSAVNCVPLEDFGGHHPDPNLTYAADLVETMKTGEHD
Cheetah_PGM1                 DAMHGVVGPYVKKILCEELGAPANSAVNCVPLEDFGGHHPDPNLTYAADLVETMKTGEHD
Crab-eating_Macaque_PGM1     DAMHGVVGPYVKKILCEELGAPANSAVNCVPLEDFGGHHPDPNLTYAADLVETMKSGEHD
Orangutan_PGM1               DAMHGVVGPYVKKILCEELGAPANSAVNCVPLEDFGGHHPDPNLTYAADLVETMKSGEHD
Rabbit_PGM1                  DAMHGVVGPYVKKILCEELGAPANSAVNCVPLEDFGGHHPDPNLTYAADLVETMKSGEHD
Mouse_PGM1                   DAMHGVVGPYVKKILCEELGAPANSAVNCVPLEDFGGHHPDPNLTYAADLVETMKSGEHD
Rat_PGM1                     DAMHGVVGPYVKKILCEELGAPANSAVNCVPLEDFGGHHPDPNLTYAADLVETMKSGEHD
Human_PGM1                   DAMHGVVGPYVKKILCEELGAPANSAVNCVPLEDFGGHHPDPNLTYAADLVETMKSGEHD
Human_PGM1
                                    α8          η2 β11      η3                α9
```

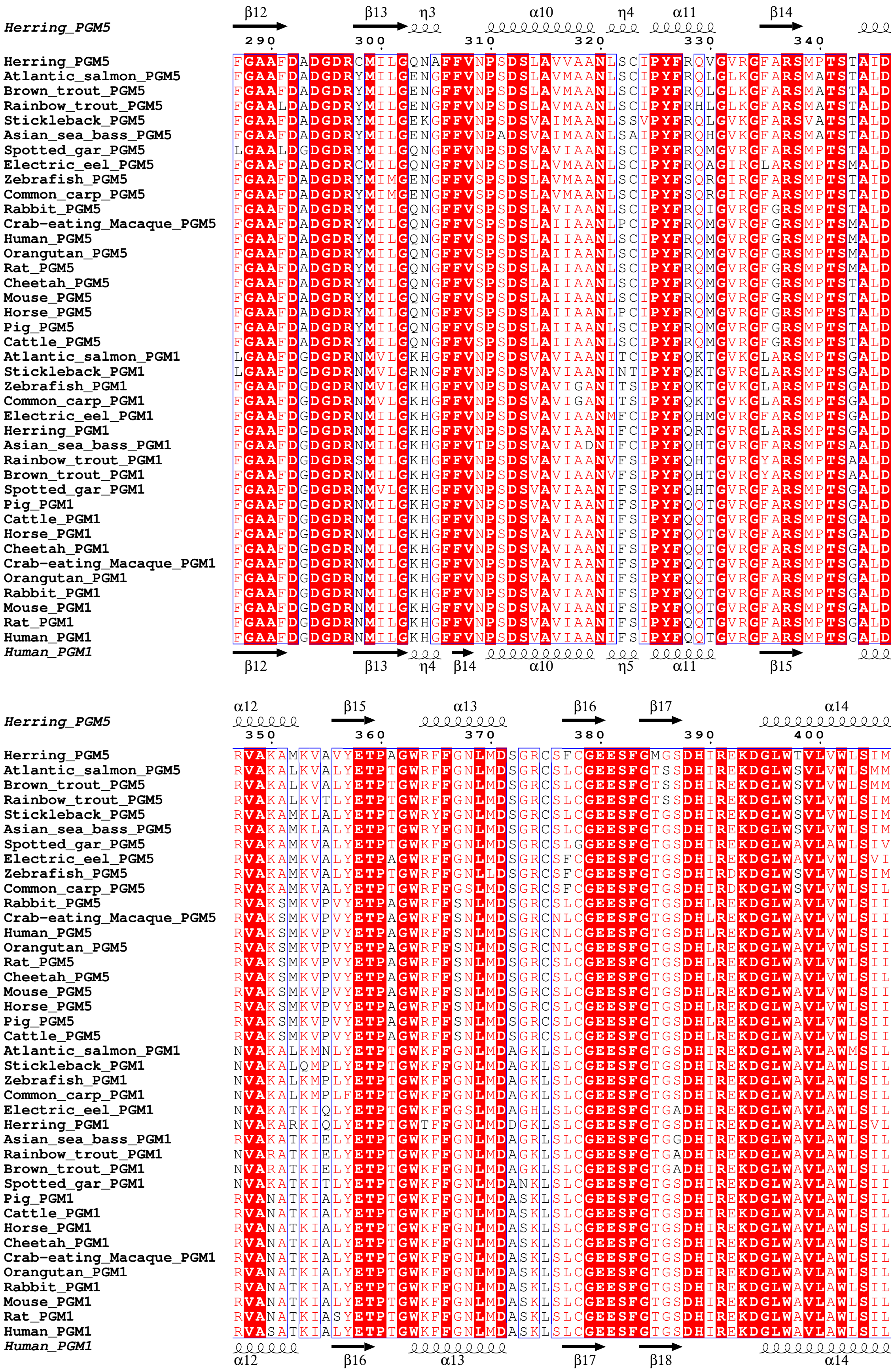

Herring_PGM5
β12
β13
η3
α10
η4
α11
β14
290
300
310
320
330
340
Herring_PGM5
Atlantic_salmon_PGM5
Brown_trout_PGM5
Rainbow_trout_PGM5
Stickleback_PGM5
Asian_sea_bass_PGM5
Spotted_gar_PGM5
Electric_eel_PGM5
Zebrafish_PGM5
Common_carp_PGM5
Rabbit_PGM5
Crab-eating_Macaque_PGM5
Human_PGM5
Orangutan_PGM5
Rat_PGM5
Cheetah_PGM5
Mouse_PGM5
Horse_PGM5
Pig_PGM5
Cattle_PGM5
Atlantic_salmon_PGM1
Stickleback_PGM1
Zebrafish_PGM1
Common_carp_PGM1
Electric_eel_PGM1
Herring_PGM1
Asian_sea_bass_PGM1
Rainbow_trout_PGM1
Brown_trout_PGM1
Spotted_gar_PGM1
Pig_PGM1
Cattle_PGM1
Horse_PGM1
Cheetah_PGM1
Crab-eating_Macaque_PGM1
Orangutan_PGM1
Rabbit_PGM1
Mouse_PGM1
Rat_PGM1
Human_PGM1
Human_PGM1
β12
β13
η4
β14
α10
η5
α11
β15
α12
β15
α13
β16
β17
α14
Herring_PGM5
350
360
370
380
390
400
Herring_PGM5
Atlantic_salmon_PGM5
Brown_trout_PGM5
Rainbow_trout_PGM5
Stickleback_PGM5
Asian_sea_bass_PGM5
Spotted_gar_PGM5
Electric_eel_PGM5
Zebrafish_PGM5
Common_carp_PGM5
Rabbit_PGM5
Crab-eating_Macaque_PGM5
Human_PGM5
Orangutan_PGM5
Rat_PGM5
Cheetah_PGM5
Mouse_PGM5
Horse_PGM5
Pig_PGM5
Cattle_PGM5
Atlantic_salmon_PGM1
Stickleback_PGM1
Zebrafish_PGM1
Common_carp_PGM1
Electric_eel_PGM1
Herring_PGM1
Asian_sea_bass_PGM1
Rainbow_trout_PGM1
Brown_trout_PGM1
Spotted_gar_PGM1
Pig_PGM1
Cattle_PGM1
Horse_PGM1
Cheetah_PGM1
Crab-eating_Macaque_PGM1
Orangutan_PGM1
Rabbit_PGM1
Mouse_PGM1
Rat_PGM1
Human_PGM1
Human_PGM1
α12
β16
α13
β17
β18
α14

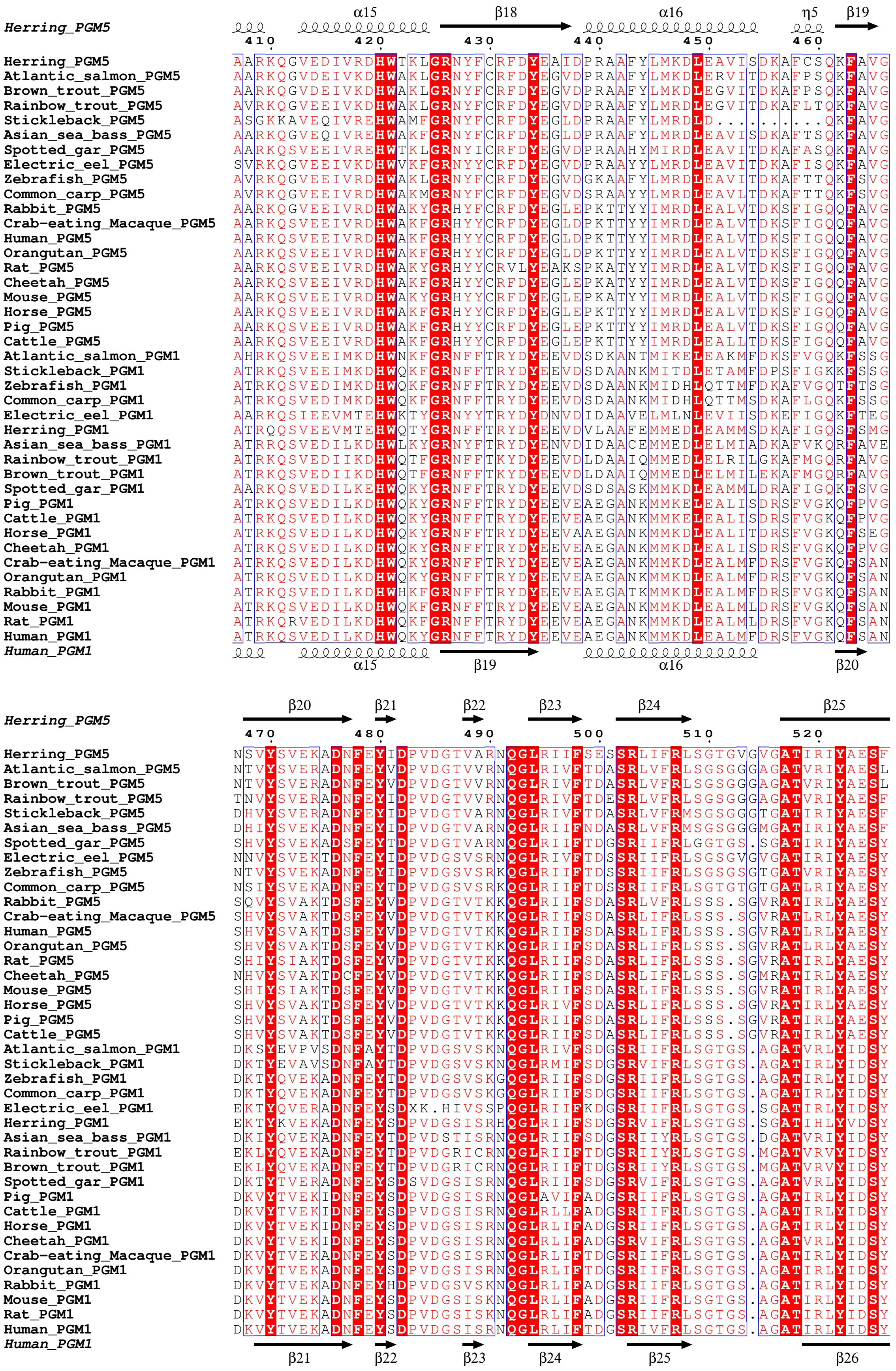
Herring_PGM5
α15 β18 α16 η5 β19
410 420 430 440 450 460
Herring_PGM5 AARKQGVEDIVRDHWTKLGRNYFCRFDYEAIDPRAAFYLMKDLEAVISDKAFCSQKFAVG
Atlantic_salmon_PGM5 AARKQGVDEIVKDHWAKLGRNYFCRFDYEGVDPRAAFYLMKDLERVITDKAFPSQKFAVG
Brown_trout_PGM5 AARKQGVDEIVKDHWAKLGRNYFCRFDYEGVDPRAAFYLMKDLEGVITDKAFPSQKFAVG
Rainbow_trout_PGM5 AVRKQGVEEIVKDHWAKLGRNYFCRFDYEGVDPRAAFYLMKDLEGVITDKAFLTQKFAVG
Stickleback_PGM5 ASGKKAVEQIVREHWAMFGRNYFCRFDYEGLDPRAAFYLMRDLD.........QKFAVG
Asian_sea_bass_PGM5 AARKQGVEQIVREHWAKFGRNYFCRFDYEGLDPRAAFYLMRDLEAVISDKAFTSQKFAVG
Spotted_gar_PGM5 AARKQSVEEIVREHWTKLGRNYICRFDYEGVDPRAAHYMIRDLEAVITDKAFASQKFAVG
Electric_eel_PGM5 SVRKQGVEEIVKDHWVKFGRNYYCRFDYEGVDPRAAYYLMRDLEAVITDKAFISQKFAVG
Zebrafish_PGM5 AVRKQGVEEIVRDHWAKLGRNYFCRFDYEGVDGKAAFYLMRDLEAVITDKAFTTQKFSVG
Common_carp_PGM5 AVRKQGVEEIVRDHWAKMGRNYFCRFDYEGVDSRAAYYLMRDLEAVLTDKAFTTQKFSVG
Rabbit_PGM5 AARKQGVEEIVRDHWAKYGRHYFCRFDYEGLEPKTTYYIMRDLEALVTDKSFIGQQFAVG
Crab-eating_Macaque_PGM5 AARKQSVEEIVRDHWAKFGRHYYCRFDYEGLDPKTTYYIMRDLEALVTDKSFIGQQFAVG
Human_PGM5 AARKQSVEEIVRDHWAKFGRHYYCRFDYEGLDPKTTYYIMRDLEALVTDKSFIGQQFAVG
Orangutan_PGM5 AARKQSVEEIVRDHWAKFGRHYYCRFDYEGLDPKTTYYIMRDLEALVTDKSFIGQQFAVG
Rat_PGM5 AARKQSVEEIVRDHWAKYGRHYYCRVLYEAKSPKATYYIMRDLEALVTDKSFIGQQFAVG
Cheetah_PGM5 AARKQSVEEIVRDHWAKFGRHYYCRFDYEGLEPKATYYIMRDLEALVTDKSFIGQQFAVG
Mouse_PGM5 AARKQSVEEIVRDHWAKYGRHYYCRFDYEGLEPKATYYIMRDLEALVTDKSFIGQQFAVG
Horse_PGM5 AARKQSVEEIVRDHWAKFGRHYYCRFDYEGLEPKTTYYIMRDLEALVTDKSFIGQQFAVG
Pig_PGM5 AARKQSVEEIVRDHWAKFGRHYYCRFDYEGLEPKTTYYIMRDLEALVTDKSFIGQQFAVG
Cattle_PGM5 AARKQSVEEIVRDHWAKFGRHYYCRFDYEGLEPKTTYYIMRDLEALLTDKSFIGQQFAVG
Atlantic_salmon_PGM1 AHRKQSVEEIMKDHWNKFGRNFFTRYDYEEVDSDKANTMIKELEAKMFDKSFVGQKFSSG
Stickleback_PGM1 ATRKQSVEEIMKDHWQKFGRNFFTRYDYEEVDSDAANKMITDLETAMFDPSFIGKKFSSG
Zebrafish_PGM1 ATRKQSVEDIMKDHWQKFGRNFFTRYDYEEVDSDAANKMIDHLQTTMFDKAFVGQTFTSG
Common_carp_PGM1 ATRKQSVEDIMKDHWQKFGRNFFTRYDYEEVDSDAANKMIDHLQTTMSDKAFLGQKFSSG
Electric_eel_PGM1 AARKQSIEEVMTEHWKTYGRNYYTRYDYDNVDIDAAVELMLNLEVIISDKEFIGQKFTEG
Herring_PGM1 ATRQQSVEEVMTEHWQTYGRNFFTRYDYEEVDVLAAFEMMEDLEAMMSDKAFIGQSFSMG
Asian_sea_bass_PGM1 ATRRQSVEDILKDHWLKYGRNYFTRYDYENVDIDAACEMMEDLELMIADKAFVKQRFAVE
Rainbow_trout_PGM1 ATRKQSVEDIIKDHWQTFGRNFFTKYDYEEVDLDAAIQMMEDLELRILGKAFMGQRFAVG
Brown_trout_PGM1 ATRKQSVEDIIKDHWQTFGRNFFTKYDYEEVDLDAAIQMMEDLELMILEKAFMGQRFAVG
Spotted_gar_PGM1 AARKQSVEDILKEHWQKYGRNFFTRYDYEEVDSDSASKMMKDLEAMMLDRAFIGQKFSVG
Pig_PGM1 ATRKQSVEDILKDHWQKYGRNFFTRYDYEEVEAEGANKMMKELEALISDRSFVGKQFPVG
Cattle_PGM1 ATRKQSVEDILKDHWQKYGRNFFTRYDYEEVEAEGANKMMKELEALISDRSFVGKQFPVG
Horse_PGM1 ATRKQSVEDILKDHWQKYGRNFFTRYDYEEVAAEGANKMMKDLEALITDRSFVGKQFSEG
Cheetah_PGM1 ATRKQSVEDILKDHWQKYGRNFFTRYDYEEVEAEGANKMMKDLEALISDRSFVGKQFPVG
Crab-eating_Macaque_PGM1 ATRKQSVEDILKDHWQKYGRNFFTRYDYEEVEAEGANKMMKDLEALMFDRSFVGKQFSAN
Orangutan_PGM1 ATRKQSVEDILKDHWQKYGRNFFTRYDYEEVEAEGANKMMKDLEALMFDRSFVGKQFSAN
Rabbit_PGM1 ATRKQSVEDILKDHWHKFGRNFFTRYDYEEVEAEGATKMMKDLEALMFDRSFVGKQFSAN
Mouse_PGM1 ATRKQSVEDILKDHWQKFGRNFFTRYDYEEVEAEGANKMMKDLEALMLDRSFVGKQFSAN
Rat_PGM1 ATRKQRVEDILKDHWQKYGRNFFTRYDYEEVEAEGANKMMKDLEALMLDRSFVGKQFSAN
Human_PGM1 ATRKQSVEDILKDHWQKYGRNFFTRYDYEEVEAEGANKMMKDLEALMFDRSFVGKQFSAN
Human_PGM1
α15 β19 α16 β20
Herring_PGM5
β20 β21 β22 β23 β24 β25
470 480 490 500 510 520
Herring_PGM5 NSVYSVEKADNFEYIDPVDGTVARNQGLRIIFSESSRLIFRLSGTGVGVGATIRIYAESF
Atlantic_salmon_PGM5 NTVYSVERADNFEYVDPVDGTVVRNQGLRIVFTDASRLVFRLSGSGGGAGATVRIYAESL
Brown_trout_PGM5 NTVYSVERADNFEYVDPVDGTVVRNQGLRIVFTDASRLVFRLSGSGGGAGATVRIYAESL
Rainbow_trout_PGM5 TNVYSVERADNFEYIDPVDGTVVRNQGLRIVFTDESRLVFRLSGSGGGAGATVRIYAESF
Stickleback_PGM5 DHVYSVERADNFEYIDPVDGTVARNQGLRIVFTDASRLVFRMSGSGGGTGATIRIYAESF
Asian_sea_bass_PGM5 DHIYSVEKADNFEYIDPVDGTVARNQGLRIIFNDASRLVFRMSGSGGGMGATIRIYAESF
Spotted_gar_PGM5 SHVYSVEKADSFEYTDPVDGTVARNQGLRIIFTDGSRIIFRLGGTGS.SGATIRIYAESY
Electric_eel_PGM5 NNVYSVEKTDNFEYIDPVDGSVSRNQGLRIVFTDSSRIIFRLSGSGVGVGATIRIYAESY
Zebrafish_PGM5 NTVYSVEKADNFEYIDPVDGSVSRKQGLRIIFTDSSRIIFRLSGSGSGTGATVRIYAESY
Common_carp_PGM5 NSIYSVEKADNFEYTDPVDGSVSRKQGLRIIFTDSSRIIFRLSGTGTGTGATLRIYAESY
Rabbit_PGM5 SQVYSVAKTDSFEYVDPVDGTVTKKQGLRIIFSDASRLVFRLSSS.SGVRATIRLYAESY
Crab-eating_Macaque_PGM5 SHVYSVAKTDSFEYVDPVDGTVTKKQGLRIIFSDASRLIFRLSSS.SGVRATLRLYAESY
Human_PGM5 SHVYSVAKTDSFEYVDPVDGTVTKKQGLRIIFSDASRLIFRLSSS.SGVRATLRLYAESY
Orangutan_PGM5 SHVYSVAKTDSFEYVDPVDGTVTKKQGLRIIFSDASRLIFRLSSS.SGVRATLRLYAESY
Rat_PGM5 SHIYSIAKTDSFEYVDPVDGTVTKKQGLRIIFSDASRLIFRLSSS.SGVRATIRLYAESY
Cheetah_PGM5 NHVYSVAKTDCFEYVDPVDGTVTKKQGLRIIFSDASRLIFRLSSS.SGMRATIRLYAESY
Mouse_PGM5 SHIYSIAKTDSFEYVDPVDGTVTKKQGLRIIFSDASRLIFRLSSS.SGVRATIRLYAESY
Horse_PGM5 SHVYSVAKTDSFEYVDPVDGTVTKKQGLRIVFSDASRLIFRLSSS.SGVRATIRLYAESY
Pig_PGM5 SHVYSVAKTDSFEYVDPVDGTVTKKQGLRIIFSDASRLIFRLSSS.SGVRATIRLYAESY
Cattle_PGM5 SHVYSVAKTDSFEYVDPVDGTVTKKQGLRIIFSDASRLIFRLSSS.SGVRATIRLYAESY
Atlantic_salmon_PGM1 DKSYEVPVSDNFAYTDPVDGSVSKNQGLRIVFSDGSRIIFRLSGTGS.AGATVRLYIDSY
Stickleback_PGM1 DKTYEVAVSDNFAYTDPVDGSVSKNQGLRMIFSDGSRVIFRLSGTGS.AGATIRLYIDSY
Zebrafish_PGM1 DKTYQVEKADNFEYTDPVDGSVSKGQGLRIIFSDGSRIIFRLSGTGS.AGATIRLYIDSY
Common_carp_PGM1 DKTYQVEKADNFEYTDPVDGSVSKGQGLRIIFSDGSRIIFRLSGTGS.AGATIRLYIDSY
Electric_eel_PGM1 EKTYQVERADNFEYSDXK.HIVSSPQGLRIIFKDGSRIIFRLSGTGS.SGATIRLYIDSY
Herring_PGM1 EKTYKVEKADNFEYSDPVDGSISRHQGLRIIFSDGSRIIFRLSGTGS.SGATIHLYIDSY
Asian_sea_bass_PGM1 DKIYQVEKADNFEYTDPVDSTISRNQGLRIIFSDGSRIIYRLSGTGS.DGATVRIYIDSY
Rainbow_trout_PGM1 EKLYQVEKADNFEYTDPVDGRICRNQGLRIIFTDGSRIIYRLSGTGS.MGATVRVYIDSY
Brown_trout_PGM1 EKLYQVEKADNFEYTDPVDGRICRNQGLRIIFTDGSRIIYRLSGTGS.MGATVRVYIDSY
Spotted_gar_PGM1 DKTYTVERADNFEYSDSVDGSISRNQGLRIIFSDGSRVIFRLSGTGS.AGATVRLYIDSY
Pig_PGM1 DKVYTVEKIDNFEYSDPVDGSISRNQGLAVIFADGSRIIFRLSGTGS.AGATIRLYIDSY
Cattle_PGM1 DKVYKVEKIDNFEYSDPVDGSISRNQGLRLLFADGSRIIFRLSGTGS.AGATIRLYIDSY
Horse_PGM1 DKVYTVEKIDNFEYSDPVDGSISRNQGLRLIFADGSRIIFRLSGTGS.AGATIRLYIDSY
Cheetah_PGM1 DKVYTVEKIDNFEYSDPVDGSISRNQGLRLIFADGSRVIFRLSGTGS.AGATVRLYIDSY
Crab-eating_Macaque_PGM1 DKVYTVEKADNFEYSDPVDGSISRNQGLRLIFTDGSRIIFRLSGTGS.AGATIRLYIDSY
Orangutan_PGM1 DKVYTVEKADNFEYSDPVDGSISRNQGLRLIFTDGSRIIFRLSGTGS.AGATIRLYIDSY
Rabbit_PGM1 DKVYTVEKADNFEYHDPVDGSVSKNQGLRLIFADGSRIIFRLSGTGS.AGATIRLYIDSY
Mouse_PGM1 DKVYTVEKADNFEYSDPVDGSISKNQGLRLIFADGSRIIFRLSGTGS.AGATIRLYIDSY
Rat_PGM1 DKVYTVEKADNFEYSDPVDGSISKNQGLRLIFADGSRIIFRLSGTGS.AGATIRLYVDSY
Human_PGM1 DKVYTVEKADNFEYSDPVDGSISRNQGLRLIFTDGSRIVFRLSGTGS.AGATIRLYIDSY
Human_PGM1
β21 β22 β23 β24 β25 β26

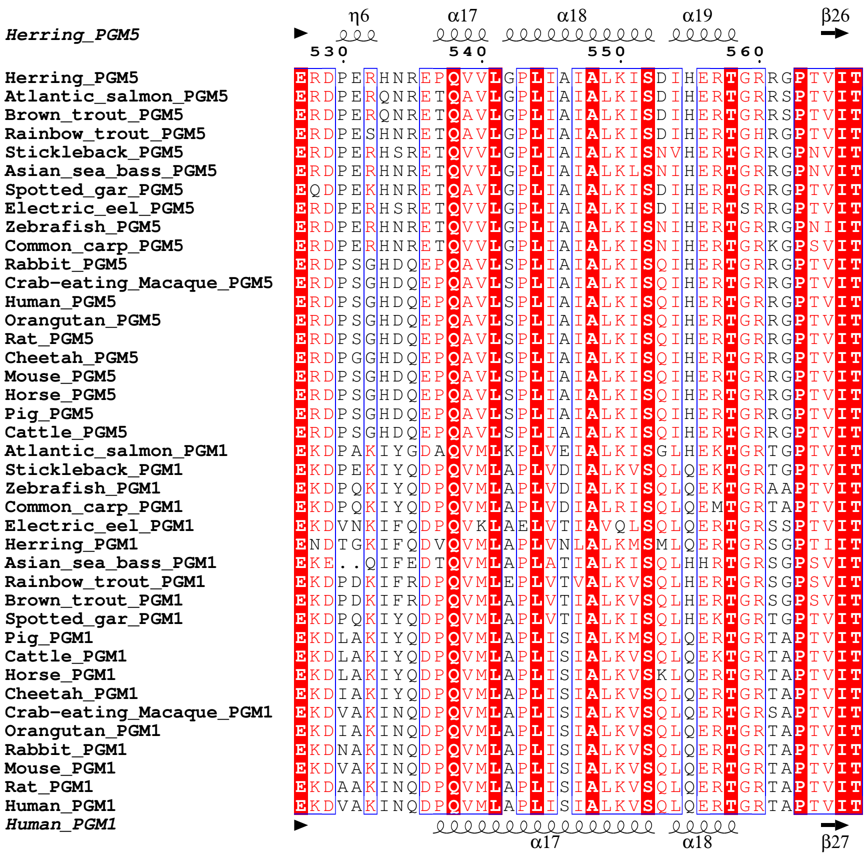
Herring_PGM5
η6 α17 α18 α19 β26
530 540 550 560
Herring_PGM5 ERDPERHNREPQVVLGPLIAIALKISDIHERTGRRGPTVIT
Atlantic_salmon_PGM5 ERDPERQNRETQAVLGPLIAIALKISDIHERTGRRSPTVIT
Brown_trout_PGM5 ERDPERQNRETQAVLGPLIAIALKISDIHERTGRRSPTVIT
Rainbow_trout_PGM5 ERDPESHNRETQAVLGPLIAIALKISDIHERTGHRGPTVIT
Stickleback_PGM5 ERDPERHSRETQVVLGPLIAIALKISNVHERTGRRGPNVIT
Asian_sea_bass_PGM5 ERDPERHNRETQVVLGPLIAIALKLSNIHERTGRRGPNVIT
Spotted_gar_PGM5 EQDPEKHNRETQAVLGPLIAIALKISDIHERTGRRGPTVIT
Electric_eel_PGM5 ERDPERHSRETQVVLGPLIAIALKISDIHERTSRRGPTVIT
Zebrafish_PGM5 ERDPERHNRETQVVLGPLIAIALKISNIHERTGRRGPNIIT
Common_carp_PGM5 ERDPERHNRETQVVLGPLIAIALKISNIHERTGRKGPSVIT
Rabbit_PGM5 ERDPSGHDQEPQAVLSPLIAIALKISQIHERTGRRGPTVIT
Crab-eating_Macaque_PGM5 ERDPSGHDQEPQAVLSPLIAIALKISQIHERTGRRGPTVIT
Human_PGM5 ERDPSGHDQEPQAVLSPLIAIALKISQIHERTGRRGPTVIT
Orangutan_PGM5 ERDPSGHDQEPQAVLSPLIAIALKISQIHERTGRRGPTVIT
Rat_PGM5 ERDPSGHDQEPQAVLSPLIAIALKISQIHERTGRRGPTVIT
Cheetah_PGM5 ERDPGGHDQEPQAVLSPLIAIALKISQIHERTGRRGPTVIT
Mouse_PGM5 ERDPSGHDQEPQAVLSPLIAIALKISQIHERTGRRGPTVIT
Horse_PGM5 ERDPSGHDQEPQAVLSPLIAIALKISQIHERTGRRGPTVIT
Pig_PGM5 ERDPSGHDQEPQAVLSPLIAIALKISQIHERTGRRGPTVIT
Cattle_PGM5 ERDPSGHDQEPQAVLSPLIAIALKISQIHERTGRRGPTVIT
Atlantic_salmon_PGM1 EKDPAKIYGDAQVMLKPLVEIALKISGLHEKTGRTGPTVIT
Stickleback_PGM1 EKDPEKIYQDPQVMLAPLVDIALKVSQLQEKTGRTGPTVIT
Zebrafish_PGM1 EKDPQKIYQDPQVMLAPLVDIALKISQLQEKTGRAAPTVIT
Common_carp_PGM1 EKDPQKIYQDPQVMLAPLVDIALRISQLQEMTGRTAPTVIT
Electric_eel_PGM1 EKDVNKIFQDPQVKLAELVTIAVQLSQLQERTGRSSPTVIT
Herring_PGM1 ENDTGKIFQDVQVMLAPLVNLALKMSMLQERTGRSGPTIIT
Asian_sea_bass_PGM1 EKE..QIFEDTQVMLAPLATIALKISQLHHRTGRSGPSVIT
Rainbow_trout_PGM1 EKDPDKIFRDPQVMLEPLVTVALKVSQLHERTGRSGPSVIT
Brown_trout_PGM1 EKDPDKIFRDPQVMLAPLVTIALKVSQLHERTGRSGPSVIT
Spotted_gar_PGM1 EKDPQKIYQDPQVMLAPLVTIALKISQLHEKTGRTGPTVIT
Pig_PGM1 EKDLAKIYQDPQVMLAPLISIALKMSQLQERTGRTAPTVIT
Cattle_PGM1 EKDLAKIYQDPQVMLAPLISIALKVSQLQEKTGRTAPTVIT
Horse_PGM1 EKDLAKIYQDPQVMLAPLISIALKVSKLQERTGRTAPTVIT
Cheetah_PGM1 EKDIAKIYQDPQVMLAPLISIALKVSQLQERTGRTAPTVIT
Crab-eating_Macaque_PGM1 EKDVAKINQDPQVMLAPLISIALKVSQLQERTGRSAPTVIT
Orangutan_PGM1 EKDIAKINQDPQVMLAPLISIALKVSQLQERTGRTAPTVIT
Rabbit_PGM1 EKDNAKINQDPQVMLAPLISIALKVSQLQERTGRTAPTVIT
Mouse_PGM1 EKDVAKINQDPQVMLAPLISIALKVSQLQERTGRTAPTVIT
Rat_PGM1 EKDAAKINQDPQVMLAPLISIALKVSQLQERTGRTAPTVIT
Human_PGM1 EKDVAKINQDPQVMLAPLISIALKVSQLQERTGRTAPTVIT
Human_PGM1
α17 α18 β27

Table S1: List of localities for herring samples included in this study. Spawning season, latitude (lat), longitude (lon), salinity and allele frequecy differences at SNPs in the vicinity of the PGM Ala330Val mutation (rs5164711).
The data are based on the previous study by Han et al. (https://www.biorxiv.org/content/10.1101/2020.07.15.204214v1) [3].

							Frequency of of PGM5 SNP					Frequency of non-PGM5 SNP	
Identifier	Sample	Location	Spawning season	lat	lon	Salinity (ppt)	rs5164711	rs5178679	rs5178707	rs5181659	rs5181660	rs5199622	rs5218152
1	A_Kalix_Baltic_Spring	Kalix	Spring	65,52	22,43	3	0,75	0,94	0,92	1,00	1,00	1	1
2	HGS1_Riga_Baltic_Spring	Gulf of Riga	Spring	58,34	24,62	5,5	0,84	0,91	0,93	1,00	1,00	1	0,98
3	HGS2_Riga_Baltic_Spring	Gulf of Riga	Spring	58,34	24,62	5,5	0,83	0,95	0,96	1,00	1,00	1	1
4	HGS3_Riga_Baltic_Autumn	Gulf of Riga	Autumn	58,1	23,92	5,5	0,65	0,73	0,75	1,00	0,97	0,75	0,84
5	HGS4_Riga_Baltic_Autumn	Gulf of Riga	Autumn	58,1	23,92	5,5	0,68	0,61	0,71	0,98	1,00	0,84	0,81
7	B_Vaxholm_Baltic_Spring	Vaxholm	Spring	59,26	18,18	6	0,71	0,84	0,92	1,00	1,00	1	1
8	PB1_Hästskär_Baltic_Spring	Hästskär	Spring	60,35	17,48	6	0,89	0,96	0,94	1,00	1,00	1	1
9	PB4_Hudiksvall_Baltic_Spring	Hudiksvall	Spring	61,45	17,3	6	0,87	0,98	1,00	1,00	1,00	0,97	1
10	PB5_Gävle_Baltic_Spring	Gävle	Spring	60,43	17,18	6	0,87	0,83	0,96	1,00	1,00	1	1
6	PB6_Gävle_Baltic_Summer	Gävle	Summer	60,43	17,18	6	0,90	0,95	0,95	1,00	1,00	1	1
11	PB7_Gävle_Baltic_Autumn	Gävle	Autumn	60,44	17,35	6	0,74	0,67	0,81	1,00	1,00	0,85	0,87
12	G_Gamleby_Baltic_Spring	Gamleby	Spring	57,5	16,27	7	0,82	0,94	0,95	1,00	1,00	1	1
13	PB11_Kalmar_Baltic_Spring	Kalmar	Spring	57,39	17,07	7	0,78	0,90	0,91	1,00	1,00	1	1
14	PB12_Karlskrona_Baltic_Spring	Karlskrona	Spring	56,1	15,33	7	0,87	0,92	0,92	1,00	1,00	1	0,97
19	HGS12_BornholmBasin_Baltic_Autumn	Bornholm Basin	Autumn	55,3	15,22	8	0,68	0,71	0,77	1,00	1,00	0,64	0,86
15	HGS71_Rugen_Baltic_Spring	Rügen	Spring	54,14	13,47	8	0,68	0,92	1,00	1,00	1,00	1	1
16	HGS72_Rugen_Baltic_Spring	Rügen	Spring	54,14	13,47	8	0,80	0,84	0,90	1,00	1,00	1	0,96
17	PN3_CentralBaltic_Baltic_Spring	Central Baltic	Spring	55,24	15,51	8	0,88	0,84	0,96	1,00	1,00	0,97	1
18	TysklandS18_Germany_Baltic	Ariadnegrund	Spring	54,22	13,58	8	0,76	0,97	0,98	1,00	1,00	1	1
21	HGS5_Schlei_Baltic_Autumn	Schlei	Autumn	54,6	9,76	9	0,69	1,00	1,00	1,00	0,96	1	1
20	HGS6_Schlei_Baltic_Spring	Schlei	Spring	54,6	9,76	9	0,67	0,74	0,80	1,00	0,97	0,93	0,89
23	HGS11_RingkobingFjord_NorthSea_Spring	Ringkobing Fjord	Spring	56,02	8,19	12	0,83	0,81	0,83	1,00	1,00	0,96	0,95
22	H_Fehmarn_Baltic_Autumn	Fehmarn	Autumn	54,5	11,3	12	0,62	0,77	0,80	0,98	0,98	0,66	0,79
24	HGS24_Landvik_Atlantic_Spring	Landvik	Spring	58,32	8,5	15	0,39	0,32	0,53	0,96	0,98	0,58	0,57
25	LandvikS17_Atlantic_Spring	Landvik	Spring	58,32	8,5	15	0,26	0,22	0,37	1,00	0,84	0,43	0,52
26	J_Traslovslage_Baltic_Spring	Träslövsläge	Spring	57,03	12,11	20	0,70	0,77	0,78	1,00	0,97	0,66	0,92
27	PB9_Kattegat_Atlantic_Spring	Kattegat, Björköfjorden	Spring	57,43	11,42	23	0,62	0,57	0,64	0,94	0,98	0,5	0,51
29	HGS8_KattegatNorth_Atlantic_Spring	Kattegat North	Spring	57,4	11,4	25	0,23	0,13	0,26	1,00	0,90	0,11	0,07

28	O_Hamburgsund_Atlantic_Spring	Hamburgsund	Spring	58,3	11,13	25	0,34	0,16	0,33	0,96	0,98	0,14	0,09
30	PB10_Skagerrak_Atlantic_Spring	Skagerrak, Brofjorden	Spring	58,19	11,21	25	0,23	0,12	0,35	1,00	1,00	0,15	0,26
31	HGS25_Lindas_Atlantic_Spring	Lindås	Spring	60,73	5,13	28	0,12	0,02	0,27	1,00	0,98	0,03	0,04
32	HGS26_Lusterfjorden_Atlantic_Spring	Lusterfjorden	Spring	61,48	7,58	32	0,15	0,00	0,33	1,00	0,97	0	0
34	HGS17_IsleOfMan_IrishSea_Autumn	Douglas Bank, Isle of Man	Autumn	54,06	-4,37	33	0,07	0,00	0,30	0,98	1,00	0	0,03
33	HGS23_Clyde_Atlantic_Spring	Ballantrae, Clyde	Spring	55,14	-5,04	33	0,03	0,02	0,24	1,00	0,97	0	0,04
35	HGS19_TeelinBay_Atlantic_Winter	Teelin Bay	Winter	54,63	-8,63	34	0,04	0,00	0,32	0,98	0,98	0	0
48	DalBoB_Atlantic_Autumn	Bonavista Bay	Autumn	48,49	-53,2	35	0,15	0,00	0,30	0,95	1,00	0	0
38	DalFB_Atlantic_Spring	Fortune Bay	Mixed	47,17	-55,38	35	0,11	0,00	0,34	0,71	0,98	0	NA
49	DalGeB_Atlantic_Autumn	German Banks	Autumn	43,16	-66,18	35	0,07	0,04	0,20	0,98	0,93	0	0
39	DalInB_Atlantic_Spring	Inner Baie Des Chaleurs	Spring	48	-65,51	35	0,13	0,00	0,43	0,96	1,00	0	0
50	DalNsF_Atlantic_Autumn	Northumberland Strait	Autumn	45,44	-62,36	35	0,16	0,00	0,29	1,00	1,00	0	0
40	DalNsS_Atlantic_Spring	Northumberland Strait	Spring	46,19	-64,09	35	0,07	0,00	0,26	0,93	0,98	0	0
36	HGS10_Downs_EnglishChannel_Winter	Downs	Winter	51,34	1,9	35	0,15	0,02	0,26	0,96	0,98	0	0,03
41	HGS15_NSSH_Atlantic_Spring	Norway	Spring	67,46	9,47	35	0,10	0,00	0,31	0,92	1,00	0	0
51	HGS16_Orkney_NorthSea_Autumn	Orkney	Autumn	59	-2	35	0,07	0,00	0,16	1,00	0,97	0	0
47	HGS18_CelticSea_Atlantic_AutumnWinter	Celtic Sea	Winter	51,59	-6,51	35	0,08	0,07	0,17	0,98	1,00	0	0
42	HGS20_CapeWrath_Atlantic_Spring	Isle of Skye	Spring	57,41	-6,13	35	0,08	0,00	0,23	0,98	0,98	0	0
46	HGS21_Hebrides_Atlantic_Mixed	West of Hebrides	Mixed	58,17	-7,23	35	0,10	0,00	0,18	1,00	0,95	0	0
52	HGS22_CapeWrath_Atlantic_Autumn	Cape Wrath	Autumn	58,61	-4,37	35	0,08	0,00	0,25	0,87	0,91	0	0
43	HGS27_Gloppen_Atlantic_Spring	Gloppen	Spring	61,77	6,16	35	0,13	0,03	0,27	1,00	1,00	0	0
37	HGS9_Greenland_Atlantic_Spring	Greenland	Summer	60,78	-47,15	35	0,13	0,00	0,03	0,93	0,98	0	0
53	N_NorthSea_Atlantic_Autumn	North Sea	Autumn	58,06	6,1	35	0,03	0,00	0,25	0,98	1,00	0	0
45	PB2_Iceland_Atlantic_Spring	Iceland, Höfn	Spring	65,49	-12,58	35	0,11	0,00	0,42	0,89	0,93	0	0
P	PB8_Pacific_Pacific_Spring	Vancouver, Strait of Georgia	Spring	NA	NA	35	0,00	0,00	0,00	1,00	0,96	0	0
44	Q_Norway_Atlantic_Atlantic_Spring	Norway	Spring	64,52	10,15	35	0,16	0,00	0,24	0,95	0,98	0	0

Table S2: Sequences of transcripts for PGM5 predicted from ENSEMBL-provided annotation of the herring genome

>ENSCHAT00000060054	MCKRYSAFDEYDYRHPLRGTSFHQLTTTPFDDQKPGTNGLRKKTTVFESKKN<br>YLQNYIQSVLSSIDLRDRQGCTMVVGSDGRYFSRTAIEVIVQMAAANGIGRL<br>VIGHNGILSTPAVSCIIRKIKAIGGIILTASRNPGGPNGDFGIKFNVANGGP<br>APDTVIDKIHQVSRTLEEYAICPDMRIDLSRLGRQDFDLENKFKPFRVEIVD<br>SVEVYLNLLRGIFDFNAIKGLLTGPDQLKMRVDAMSGVMGPYVRRILCDELG<br>APANSAVNCVPLEDFGGHYPDPNLTYATGLVDAMKGGEFGFGAAFDADGDRC<br>MILGQNAFFVNPSDSLAVVAANLSCIPYFRQVGVRGFARSMPTSTAIDRVAK<br>AMKVAVYETPAGWRFFGNLMDSGRCSFCGEESFGMGSDHIREKDGLWTVLVW<br>LSIMAARKQGVEDIVRDHWTKLGRNYFCRFDYEAIDPRAAFYLMKDLEAVIS<br>DKAFCSQKFAVGNSVYSVEKADNFEYIDPVDGTVARNQGLRIIFSESSRLIF<br>RLSGTGVGVGATIRIYAESFERDPERHNREPQVVLGPLIAIALKISDIHERT<br>GRRGPTVIT
>ENSCHAT00000060060	YEVYREFLSATIAKTNPIPVVTVQTTPFDDQKPGTNGLRKKTTVFESKKNYL<br>QNYIQSVLSSIDLRDRQGCTMVVGSDGRYFSRTAIEVIVQMAAANGIGRLVI<br>GHNGILSTPAVSCIIRKIKAIGGIILTASRNPGGPNGDFGIKFNVANGGPAP<br>DTVIDKIHQVSRTLEEYAICPDMRIDLSRLGRQDFDLENKFKPFRVEIVDSV<br>EVYLNLLRGIFDFNAIKGLLTGPDQLKMRVDAMSGVMGPYVRRILCDELGAP<br>ANSAVNCVPLEDFGGHYPDPNLTYATGLVDAMKGGEFGFGAAFDADGDRCMI<br>LGQNAFFVNPSDSLAVVAANLSCIPYFRQVGVRGFARSMPTSTAIDRVAKAM<br>KVAVYETPAGWRFFGNLMDSGRCSFCGEESFGMGSDHIREKDGLWTVLVWLS<br>IMAARKQGVEDIVRDHWTKLGRNYFCRFDYEAIDPRAAFYLMKDLEAVISDK<br>AFCSQKFAVGNSVYSVEKADNFEYIDPVDGTVARNQGLRIIFSESSRLIFRL<br>SGTGVGVGATIRIYAESFERDPERHNREPQVVLGPLIAIALKISDIHERTGR<br>RGPTVIT
>ENSCHAT00000060079	METNPIPVVTVQTTPFDDQKPGTNGLRKKTTVFESKKNYLQNYIQSVLSSID<br>LRDRQGCTMVVGSDGRYFSRTAIEVIVQMAAANGIGRLVIGHNGILSTPAVS<br>CIIRKIKAIGGIILTASRNPGGPNGDFGIKFNVANGGPAPDTVIDKIHQVSR<br>TLEEYAICPDMRIDLSRLGRQDFDLENKFKPFRVEIVDSVEVYLNLLRGIFD<br>FNAIKGLLTGPDQLKMRVDAMSGVMGPYVRRILCDELGAPANSAVNCVPLED<br>FGGHYPDPNLTYATGLVDAMKGGEFGFGAAFDADGDRCMILGQNAFFVNPSD<br>SLAVVAANLSCIPYFRQVGVRGFARSMPTSTAIDRVAKAMKVAVYETPAGWR<br>FFGNLMDSGRCSFCGEESFGMGSDHIREKDGLWTVLVWLSIMAARKQGVEDI<br>VRDHWTKLGRNYFCRFDYEAIDPRAAFYLMKDLEAVISDKAFCSQKFAVGNS<br>VYSVEKADNFEYIDPVDGTVARNQGLRIIFSESSRLIFRLSGTGVGVGATIR<br>IYAESFERDPERHNREPQVVLGPLIAIALKISDIHERTGRRGPTRRAFTKCR<br>VLFFSFFYYYCYYFNISRHH
>ENSCHAT00000060097	METNPIPVVTVQTTPFDDQKPGTNGLRKKTTVFESKKNYLQNYIQSVLSSID<br>LRDRQGCTMVVGSDGRYFSRTAIEVIVQMAAANGIGRLVIGHNGILSTPAVS

	CIIRKIKAIGGIILTASRNPGGPNGDFGIKFNVANGGPAPDTVIDKIHQVSR TLEEYAICPDMRIDLSRLGRQDFDLENKFKPFRVEIVDSVEVYLNLLRGIFD FNAIKGLLTGPDQLKMRVDAMSGVMGPYVRRILCDELGAPANSAVNCVPLED FGGHYPDPNLTYATGLVDAMKGGEFGFGAAFDADGDRCMILGQNAFFVNPSD SLAVVAANLSCIPYFRQVGVRGFARSMPTSTAIDRVAKAMKVAVYETPAGWR FFGNLMDSGRCSFCGEESFGMGSDHIREKDGLWTVLVWLSIMAARKQGVEDI VRDHWTKLGRNYFCRFDYEAIDPRAAFYLMKDLEAVISDKAFCSQKFAVGNS VYSVEKADNFEYIDPVDGTVARNQGLRIIFSESSRLIFRLSGTGVGVGATIR IYAESFERDPERHNREPQVQDTTLTLGLSSSFSSSSSASFSSSFVHTASPDY FAQMSESKTTQFMN

Table S3: Sequences of aPGM5 and bPGM5 used in this manuscript

>pNIC28-Bsa4_aPGM5_Ala330_Atlantic_aa	>pNIC28-Bsa4_bPGM5_Val330_Baltic_aa
MHHHHHHSSGVDLGTENLYFQ↓S	MHHHHHHSSGVDLGTENLYFQ↓S
METNPIPVVTVQTTPFDDQKPGTNGLRKKTTVFES	METNPIPVVTVQTTPFDDQKPGTNGLRKKTTVFES
KKNYLQNYIQSVLSSIDLRDRQGCTMVVGSDGRYF	KKNYLQNYIQSVLSSIDLRDRQGCTMVVGSDGRYF
SRTAIEVIVQMAAANGIGRLVIGHNGILSTPAVSC	SRTAIEVIVQMAAANGIGRLVIGHNGILSTPAVSC
IIRKIKAIGGIILTASRNPGGPNGDFGIKFNVANG	IIRKIKAIGGIILTASRNPGGPNGDFGIKFNVANG
GPAPDTVIDKIHQVSRTLEEYAICPDMRIDLSRLG	GPAPDTVIDKIHQVSRTLEEYAICPDMRIDLSRLG
RQDFDLENKFKPFRVEIVDSVEVYLNLLRGIFDFN	RQDFDLENKFKPFRVEIVDSVEVYLNLLRGIFDFN
AIKGLLTGPDQLKMRVDAMSGVMGPYVRRILCDEL	AIKGLLTGPDQLKMRVDAMSGVMGPYVRRILCDEL
GAPANSAVNCVPLEDFGGHYPDPNLTYATGLVDAM	GAPANSAVNCVPLEDFGGHYPDPNLTYATGLVDAM
KGGEFGFGAAFDADGDRCMILGQNAFFVNPSDSLA	KGGEFGFGAAFDADGDRCMILGQNAFFVNPSDSLA
VVAANLSCIPYFRQ**A**GVRGFARSMPTSTAIDRVAK	VVAANLSCIPYFRQ**V**GVRGFARSMPTSTAIDRVAK
AMKVAVYETPAGWRFFGNLMDSGRCSFCGEESFGM	AMKVAVYETPAGWRFFGNLMDSGRCSFCGEESFGM
GSDHIREKDGLWTVLVWLSIMAARKQGVEDIVRDH	GSDHIREKDGLWTVLVWLSIMAARKQGVEDIVRDH
WTKLGRNYFCRFDYEAIDPRAAFYLMKDLEAVISD	WTKLGRNYFCRFDYEAIDPRAAFYLMKDLEAVISD
KAFCSQKFAVGNSVYSVEKADNFEYIDPVDGTVAR	KAFCSQKFAVGNSVYSVEKADNFEYIDPVDGTVAR
NQGLRIIFSESSRLIFRLSGTGVGVGATIRIYAES	NQGLRIIFSESSRLIFRLSGTGVGVGATIRIYAES
FERDPERHNREPQVVLGPLIAIALKISDIHERTGR	FERDPERHNREPQVVLGPLIAIALKISDIHERTGR
RGPTVIT*	RGPTVIT*

Table S4: RMSD values from pairwise structure comparison using PDBeFOLD [4]. hsPGM1-2 = human PGM1 isoform 2.

**Structure**	**PDB code**	**Chain**	**aPGM5 apo 6Y8X, Chain A** RMSD (Å) (aligned Cα atoms)	**bPGM5 apo 6Y8Z, Chain A** RMSD (Å) (aligned Cα atoms)	**bPGM5 + G-1-P 6Y8Y, Chain A** RMSD (Å) (aligned Cα atoms)
**aPGM5 apo**	6Y8X	A	0	0.641 (545)	0.610 (547)
**bPGM5 apo**	6Y8Z	A	0.641 (545)	0	0.267 (567)
**bPGM5 + G-1-P**	6Y8Y	A	0.610 (547)	0.267 (567)	0
**hsPGM1 apo**	5EPC	A	1.872 (532)	1.538 (540)	1.656 (540)
**hsPGM1 apo**	5EPC	B	1.663 (529)	1.362 (539)	1.473 (536)
**hsPGM1 + G-6-P**	6BJ0	A	1.789 (533)	1.464 (540)	1.591 (542)
**hsPGM1 + G-6-P**	6BJ0	B	1.721 (534)	1.378 (540)	1.508 (544)
**ocPGM1 apo**	3PMG	A	1.582 (481)	1.893 (527)	1.654 (505)
**ocPGM1 apo**	3PMG	B	1.899 (538)	1.514 (543)	1.645 (545)
**ptPGM + sulfate**	1KFI	A	1.187 (522)	1.169 (531)	1.181 (534)
**ptPGM + sulfate**	1KFI	B	1.748 (520)	1.493 (525)	1.634 (530)
**ptPGM apo**	1KFQ	A	1.845 (467)	1.911 (493)	1.891 (484)
**ptPGM apo**	1KFQ	B	1.716 (477)	1.831 (503)	1.872 (499)
**hsPGM1-2 apo**	6SNP	A	1.304 (530)	1.064 (539)	1.139 (537)
**hsPGM1-2 + G-1-P**	6SNO	A	1.307 (534)	1.000 (540)	1.074 (544)
**hsPGM1-2 + G-6-P**	6SNQ	A	1.366 (523)	1.109 (539)	1.188 (539)
**ocPGM1 + G-1-P-6-V ***	1C4G	A	1.100 (465)	1.285 (491)	1.284 (487)
**ocPGM1 + G-1-P-6-V ***	1C4G	B	1.960 (517)	1.664 (534)	1.802 (536)
**ocPGM1 + G-1,6-BP ***	1C47	A	1.542 (495)	1.733 (530)	1.550 (511)
**ocPGM1 + G-1,6-BP ***	1C47	B	1.932 (529)	1.593 (533)	1.706 (533)

* Structures have serious errors in placement of domain 4.

Table S5: Details of sequences included in Figure S8. The query coverage was 98-100%.

Species common name	Species latin name	PGM1 from species			PGM5 from species		
		Accession code UniProt or GenBank	Sequence identity to bPGM5 (%)	Sequence identity to hsPGM1 (%)	Accession code UniProt or GenBank	Sequence identity to bPGM5 (%)	Sequence identity to hsPGM1 (%)
Herring (baltic)	*Clupea harengus (membras)*	XP_012673001.1	67.7	76.3	XP_012676095.1	100	67.6
Zebrafish	*Danio rerio*	F1QF00	64.1	82.0	B0R0B3	86.2	66.4
Stickleback	*Gasterosteus aculeatus*	G3NVH9	63.0	79.9	G3NVP3	82.7	63.4
Spotted gar	*Lepisosteus oculatus*	XP_006634913.2	70.7	83.8	W5MSS7	85.4	70.4
Common carp	*Cyprinus carpio*	XP_018950613.1	65.1	81.1	XP_018977408.1	85.4	65.8
Atlantic salmon	*Salmo salar*	B5DG72	62.9	77.9	A0A1S3PFY1	85.71	64.8
Rainbow trout	*Oncorhynchus mykiss*	XP_021468451.1	68.3	75.2	XP_021477693.1	85.2	64.2
Electric eel	*Electrophorus electricus*	XP_026854191.1	66.8	74.2	XP_026870188.1	87.3	66.7
Brown trout	*Salmo trutta*	XP_029559985.1	68.3	75.9	XP_029572366.1	85.5	65.6
Asian sea bass / barramundi	*Lates calcarifer*	XP_018532842.1	68.8	76.3	XP_018525124.1	86.8	65.1
Human	*Homo sapiens*	P36871	67.6	100	Q15124	78.7	65.1
Rabbit	*Oryctolagus cuniculus*	P00949	68.3	97.0	G1T6S2	79.23	65.8
Mouse	*Mus musculus*	Q9D0F9	68.2	97.3	Q8BZF8	78.9	65.1

Sumatran Orangutan	*Pongo abelii*	K7EU15	67.8	99.3	A0A2J8XHD5	78.5	65.1
Crab-eating Macaque	*Macaca fascicularis*	Q4R5E4	67.8	98.8	A0A2K5VRN8	78.5	65.0
Horse	*Equus caballus*	F6X8Q2	68.3	96.4	F7DQ57	78.7	65.3
Cheetah	*Acinonyx jubatus*	XP_026914801.1	68.9	96.1	XP_026896886.1	79.2	65.5
Pig	*Sus scrofa*	F1S814	68.3	96.1	F1SJE6	79.1	65.3
Cattle	*Bos taurus*	Q08DP0	68.2	96.1	A6QNJ7	78.9	65.1
Rat	*Rates norvegicus*	P38652	68.0	96.6	D3ZVR9	78.4	64.6
Ciliate PGM	*Paramecium tetraurelia*	P47244	47.0	50.5			
Human (isoform 2)	*Homo sapiens*	P36871-2	69.7	93.1			

REFERENCES

1. Laskowski, R.A.; Swindells, M.B. LigPlot+: multiple ligand-protein interaction diagrams for drug discovery. *J Chem Inf Model* **2011**, *51*, 2778–2786, doi:10.1021/ci200227u.

2. Robert, X.; Gouet, P. Deciphering key features in protein structures with the new ENDscript server. *Nucleic Acids Res.* **2014**, *42*, W320-4, doi:10.1093/nar/gku316.

3. Han, F.; Jamsandekar, M.; Pettersson, M.E.; Su, L.; Fuentes-Pardo, A.; Davis, B.; Bekkevold, D.; Berg, F.; Cassini, M.; Dahle, G.; et al. The genetic architecture underlying ecological adaptation in Atlantic herring is not consistent with the infinitesimal model. *bioRxiv* **2020**, 2020.07.15.204214, doi:10.1101/2020.07.15.204214.

4. Krissinel, E.; Henrick, K. Secondary-structure matching (SSM), a new tool for fast protein structure alignment in three dimensions. *Acta Crystallogr. Sect. D Biol. Crystallogr.* **2004**, *60*, 2256–2268, doi:10.1107/S0907444904026460.
